# Comparative mitochondrial genomics of snakes: extraordinary substitution rate dynamics and functionality of the duplicate control region

**DOI:** 10.1186/1471-2148-7-123

**Published:** 2007-07-26

**Authors:** Zhi J Jiang, Todd A Castoe, Christopher C Austin, Frank T Burbrink, Matthew D Herron, Jimmy A McGuire, Christopher L Parkinson, David D Pollock

**Affiliations:** 1 Department of Biological Sciences, Biological Computation and Visualization Center, Louisiana State University, Baton Rouge, LA, USA; 2 Computational Biology, Scripps Florida, Jupiter, FL, USA; 3 Department of Biology, University of Central Florida, Orlando, FL, USA; 4 Department of Biochemistry and Molecular Genetics, University of Colorado Health Sciences Center, Aurora, CO, USA; 5 Museum of Natural Science, Louisiana State University, Baton Rouge, LA, USA; 6 Department of Biology, City University of New York, Staten Island, USA; 7 Department of Ecology and Evolutionary Biology, University of Arizona, Tucson, AZ, USA; 8 Department of Integrative Biology, University of California, Berkeley, Berkeley, CA, USA; 9 Department of Biochemistry and Molecular Genetics, University of Colorado Health Sciences Center, Aurora, CO, USA

## Abstract

**Background:**

The mitochondrial genomes of snakes are characterized by an overall evolutionary rate that appears to be one of the most accelerated among vertebrates. They also possess other unusual features, including short tRNAs and other genes, and a duplicated control region that has been stably maintained since it originated more than 70 million years ago. Here, we provide a detailed analysis of evolutionary dynamics in snake mitochondrial genomes to better understand the basis of these extreme characteristics, and to explore the relationship between mitochondrial genome molecular evolution, genome architecture, and molecular function. We sequenced complete mitochondrial genomes from Slowinski's corn snake (*Pantherophis slowinskii*) and two cottonmouths (*Agkistrodon piscivorus*) to complement previously existing mitochondrial genomes, and to provide an improved comparative view of how genome architecture affects molecular evolution at contrasting levels of divergence.

**Results:**

We present a Bayesian genetic approach that suggests that the duplicated control region can function as an additional origin of heavy strand replication. The two control regions also appear to have different intra-specific versus inter-specific evolutionary dynamics that may be associated with complex modes of concerted evolution. We find that different genomic regions have experienced substantial accelerated evolution along early branches in snakes, with different genes having experienced dramatic accelerations along specific branches. Some of these accelerations appear to coincide with, or subsequent to, the shortening of various mitochondrial genes and the duplication of the control region and flanking tRNAs.

**Conclusion:**

Fluctuations in the strength and pattern of selection during snake evolution have had widely varying gene-specific effects on substitution rates, and these rate accelerations may have been functionally related to unusual changes in genomic architecture. The among-lineage and among-gene variation in rate dynamics observed in snakes is the most extreme thus far observed in animal genomes, and provides an important study system for further evaluating the biochemical and physiological basis of evolutionary pressures in vertebrate mitochondria.

## Background

The vertebrate mitochondrial (mt) genome has been an important model system for studying molecular evolution, organismal phylogeny, and genome structure. Despite extensive molecular studies, little is known regarding the ways in which genome architecture might affect the various aspects of genome function and evolution (including replication, transcription, and RNA/protein function, as well as rates and patterns of nucleotide evolution). Nevertheless, patterns linking mt genome structure, function, and nucleotide evolution have begun to emerge [1-3].

Among the most direct demonstrated links among genome architecture, function and nucleotide evolution is that relating the asymmetrical genome replication process with gradients of transition substitutions in vertebrate mitochondrial genomes [1-3]. Gradients of transition mutations, arising from deamination mutations, are observed due to the differential time regions of the mt genome spend in an asymmetric mutagenic state during genome replication (*T*_*AMS *_alternatively referred to as the time spent in a single-stranded state, *D*_*SSH*_, [4-6], but there is some controversy about this: see Additional file 1). Thus, gradients of transition biases are dependent upon the relative position of the functional origins of heavy and light strand replication. In vertebrate mt genomes, the origin of heavy strand replication (O_H_) is thought to be within the control region (CR), and the origin of light strand replication (O_L_) in the tRNA cluster referred to as the WANCY region (named for the five amino acids coded for by these five tRNAs). Among transition classes in vertebrate mt genomes, T→C light strand substitutions at degenerate 3^rd ^codon positions increase linearly with increasing *T*_*AMS *_and C/T nucleotide frequencies at degenerate 3^rd ^positions are good predictors of *T*_*AMS *_[4].

The mt genomes of snakes contain a number of characteristics that are unusual among vertebrates, and represent an ideal model for exploring potential links among genome structure, function, and evolution. Snake mitochondrial genomes appear to have the highest evolutionary rates among vertebrates and contain truncated tRNAs and other shortened genes [7,8]. All snake species sampled to date, except the scolecophidian snakes *Leptotyphlops dulcis, Ramphotyphlops australis*, and *Typhlops murius*, have a duplicated control region (CR2) between NADH dehydrogenase subunit 1 (ND1) and subunit 2 (ND2), in addition to a control region (CR1) adjacent to the 5'-end of the 12s rRNA as it is in other vertebrates [7-11]. These two control regions appear to undergo concerted evolution that acts to homogenize the nucleotide sequence of each duplicate copy within a given genome [7-9]. The functionality of these two control regions in transcription and initiation of heavy strand replication is not clear, but given that the nucleotide sequence of each is nearly identical, any functional features that are not dependent on surrounding sequences should be similar. In contrast, recent evidence suggests that initiation of heavy strand replication may be distributed across a broad zone, including cytochrome b (CytB) and NADH dehydrogenase subunit 6 (ND6) [12], indicating that CR2 may not function as effectively in this role.

A number of interesting questions arise that might be addressed through comparative analysis, including: (1) does one or the other, or do both control regions function as origins of heavy strand DNA synthesis? (2) does the altered genome structure affect patterns of snake mt genome molecular evolution? (3) when during snake evolution did various features arise, and were any changes synchronous? (4) do patterns of mt molecular evolution vary at different depths of phylogeny? and (5) is there any evidence or plausible rationale for selection as a causative agent in generating differences in genomic structure and molecular evolutionary patterns?

To investigate outstanding questions regarding snake mitochondrial genome evolution, structure, and function, we analyzed a dataset consisting of three new complete snake mitochondrial genomes together with all eight previously published snake mitochondrial genomes that were available at the time of this study, and 42 other vertebrate mitochondrial genomes for comparative purposes. The new snake genomes were obtained from one *Pantherophis slowinskii *(Colubroidea: Colubridae; a corn snake from Louisiana; previously *Elaphe guttata*), and from two *Agkistrodon piscivorus *(Colubroidea: Viperidae; the cottonmouth or water moccasin; one specimen from Florida and the other from Louisiana).

## Results

### Brief summary of the new complete snake mitochondrial genomes

**Figure 1:**
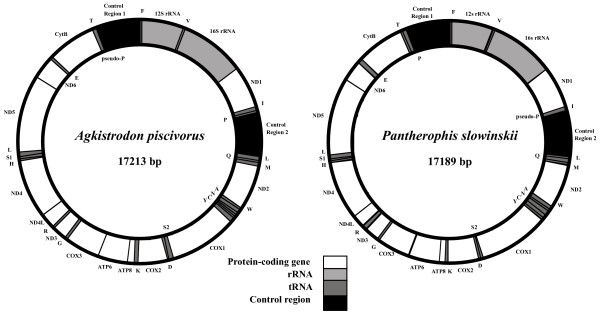
**Annotated mitochondrial genome maps of *Agkistrodon piscivorus *and *Pantherophis slowinskii***. The two *A. piscivorus *samples (*Api1 *and *Api2*) have identical annotations except for minor variations in gene length. Labels of genes outside the circle refer to genes transcribed from the light strand, and names within the circle represent genes transcribed from the heavy strand.

The gene contents of *A. piscivorus *and *P. slowinskii *mt genomes are very similar to other snakes (Figure 1; for detailed genome annotation see Additional file 2). As in all known alethinophidian snake mt genomes, these species have a presumably duplicated control region (CR2) between ND1 and ND2, in addition to the original control region (CR1) present in all vertebrates adjacent to the 5' end of the 12s rRNA gene [7-9]. These genomes also possess the translocated tRNA^Leu ^common to all alethinophidian snakes sampled thus far (3' of CR2). In addition to an intact tRNA^Pro ^between CytB and CR1, *P. slowinskii *has an apparent pseudo-tRNA^Pro ^gene (Ψ-tRNA^Pro^) between ND1 and CR2 (as does the previously sequenced colubrid, *Dinodon semicarinatus*). In *P. slowinskii*, this Ψ-tRNA^Pro ^exactly matches the first 35 bases of tRNA^Pro^. In contrast, the intact tRNA^Pro ^of *A. piscivorus *(and the only previously sequenced viperid, *Ovophis okinavensis*) is located between ND1 and CR2 (exactly the location of Ψ-tRNA^Pro ^in the colubrids), and there is a 31 bp non-coding fragment between tRNA^Thr ^and CR1, where tRNA^Pro ^is usually located. In *O. okinavensis*, this is clearly a Ψ-tRNA^Pro^, since these 31 bp are an exact match to the CR1-proximal end of the complete tRNA^Pro^, but in *A. piscivorus *the homology is much less clear. These alternative positions of tRNA^Pro^, Ψ-tRNA^Pro^, and a previously noted [7] duplication of tRNA^Phe ^in *O. okinavensis *are the only known mt genome gene rearrangements identified within alethinophidian snakes.

Within a mt genome, the two copies of the CR in each newly sequenced species are nearly identical (e.g., *Api1 *CR1 and CR2), as is typical for alethinophidian snakes [7,8]. In *P. slowinskii *there is a single point mutation and four extra nucleotides at one end of CR1, in *Api1 *there is one indel plus 14 extra nucleotides on one end of CR1, and in *Api2 *there are seven indels and two base changes between the two control regions. Between *Api1 *and *Api2*, CR1 differs by five indels and 19 point mutations, whereas CR2 differs by three indels (two at the 5' end) and 18 point mutations.

### Comparison of *Agkistrodon piscivorus *genomes

**Figure 2:**
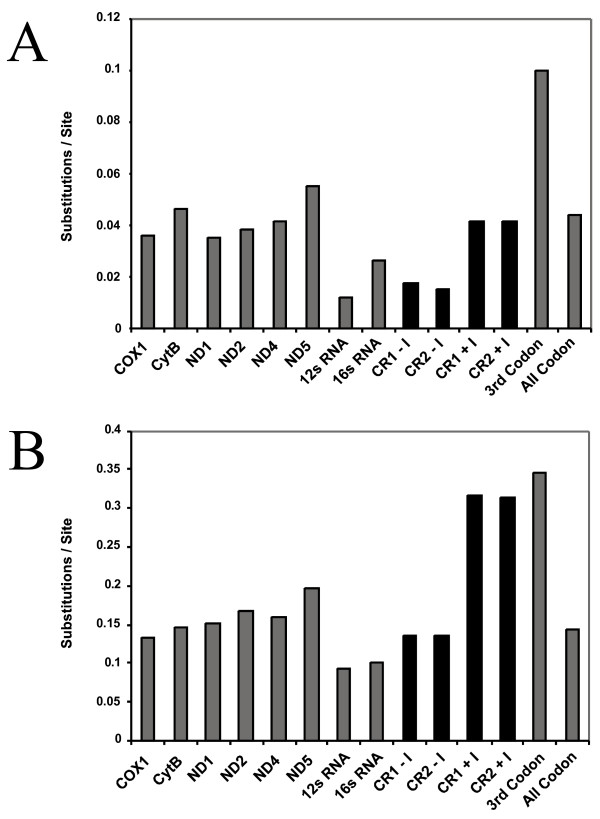
**Differences per site for homologous genes or groups of sites in the two *Agkistrodon piscivorus *genomes and in the two viperid genomes **. The differences per site are shown for a comparison of *Api1 *and *Api2 *(A), and for *A. piscivorus *(mean of *Api1 *and *Api2*) and *Ovophis okinavensis *(B). Differences are shown only for the longer protein-coding genes. For the control regions only (shaded black), differences are shown for each aligned site including indels (e.g., CR1+I), or excluding indels (e.g., CR1-I). For all other genes, indels are not included in the difference measure. The bars for 3^rd ^codon positions (3 rd Codon) and for all codon positions (All Codon) are summed over all protein-coding genes.

Polymorphisms were observed between the two *A. piscivorus *genomes, *Api1 *and *Api2*, for all protein and rRNA genes and for 14 of 22 tRNAs (see Additional file 2). The 12s and 16s rRNAs were the most conserved genes between the two *A. piscivorus *individuals, with 2% and 3% sequence divergence respectively (Figure 2A; Additional file 2). Protein-coding genes differed more, up to 6.2% for ND3 (Figure 2A; Additional file 2). Most differences occurred at 3^rd ^codon positions (Figure 2A; Additional file 2), as expected under predominantly neutral patterns of divergence (for example, 57/58 substitutions in COX1 were at 3^rd ^codon positions).

Within *A. piscivorus*, the control regions (e.g., CR1 in *Api1 *vs. CR1 in *Api2*) are as similar to each other as are the rRNA genes, and more similar than the protein coding genes (Figure 2A). This is in strong contrast to the normal pattern of divergence between vertebrate species, for which control region similarity is far less than that of protein-coding or rRNA genes, e.g., [13,14]. Between *A. piscivorus *and the other viperid, *O. okinavensis*, the control regions have 30% more differences (with indels included) than the rRNAs, and are on par with divergence in the protein-coding genes (Figure 2B). If indels are included, the control regions of these two species are nearly as different as the average 3^rd ^codon position (Figure 2B). The high degree of similarity (low divergence) observed between the CRs of the two *A. piscivorus *individuals is surprising, and contrasts sharply with the high relative divergence of CRs between *O. okinavensis *and *A. piscivorus *(Figure 2).

### Phylogenetics

**Figure 3:**
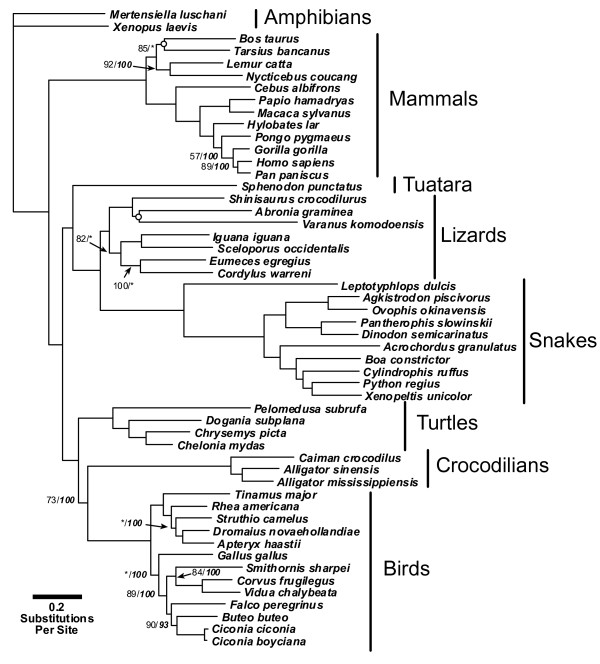
**Maximum likelihood phylogeny for vertebrate taxa included in this study**.This phylogeny is based on all protein-coding and rRNA genes. Most branches have greater than 95% support for both NJ ML distance bootstrap and Bayesian posterior probability support (see Methods), and are not annotated with support values. Where support from either measure is less than 95%, the support values are indicated by ratios, with the ML bootstrap support on top and the Bayesian posterior probability support below in italics, except for two nodes with less than 50% support by either measure, which are indicated by a hollow circle. Other than for these two nodes, support values less than 50% are indicated with an asterisk (*).

We present the phylogenetic tree estimate obtained by ML, with NJ bootstrap values (BS) and posterior probabilities (PP) for nodal support, which were generally high (Figure 3). Our phylogeny estimate provides a well-resolved and, in many cases, strongly-supported amniote phylogeny that is consistent with previous molecular studies. Differences between the ML topology (Figure 3), and the topology based on Bayesian analysis (not shown) were minor, and included an alternative placement of *Bos taurus *among mammals, and alternative placements of *Gallus gallus *and *Rhea americana *among birds. Additionally, relationships among lizard taxa varied, with *Cordylus warreni *estimated to be the sister lineage to all other lizards, and an alternative placement of *Varanus komodoensis*.

**Figure 4:**
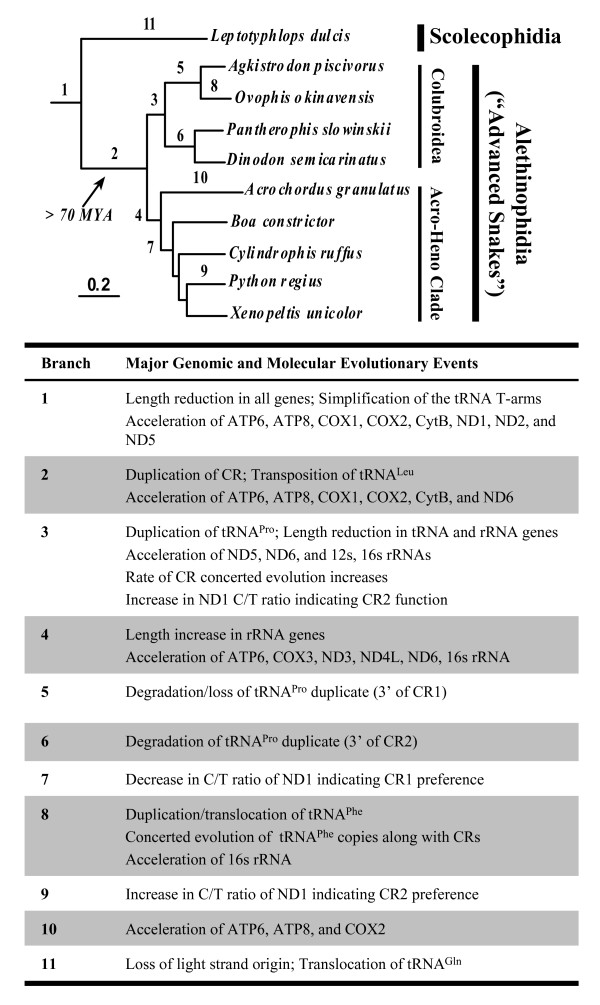
**Hypotheses for the relative timing of alterations in mitochondrial genome architecture and molecular evolution throughout snake phylogeny**. The topological relationships among snakes and branch lengths shown are the same as in Figure 3. Major groups of snakes are indicated along with the approximate diversification time of the Alethinophidia.

All phylogenetic estimates provided an identical, well-supported topology for relationships among snakes (Figure 3), and a summary of results concerning snake relationships is shown in Figure 4. The Scolecophidia (Typhlopoidea), represented here by *L. dulcis*, formed the sister group to all remaining snakes. Rather than finding support for the Caenophidia (*Acrochordus *plus Colubroidea [7,15]), we find strong support for *Acrochordus *as the sister lineage to the Henophidia (including *Cylindrophis*). Hereafter we will therefore operationally refer to this clade including the Henophidia and *Acrochordus *as the "Acro-Heno" clade, and we will refer to its sister clade as the Colubroidea [16].

The snake and overall amniote phylogeny are strongly supported by our analysis of this dataset, and we henceforth treat this phylogeny as accurate. We wish to emphasize, however, that the consistency of the phylogenetic results do not guarantee that they are, in fact, accurate. Although for simplicity we present a single nucleotide substitution model for the entire dataset we have analyzed an expanded version of this dataset (with additional unpublished snake and lizard mt genomes; with and without inclusion of the rRNA genes) using complex partitioned models for each gene and codon position. The results of this expanded (highly partitioned-model) phylogeny estimate (not shown) were essentially identical to those presented here in terms of the placement of snakes within squamates, and relationships among squamates. We provide evidence below for extremely complex non-stationary patterns of nucleotide substitution across branches and mt genome regions, and have previously identified asymmetric substitution gradients in mt genomes [4] that may vary among species (e.g., primates [3]). These latter patterns cannot be modeled using available phylogenetic programs (e.g., MrBayes [17]). We expect our phylogenetic estimates here to represent a good estimate of the relationships among mt genomes sampled, and if minor inaccuracies in the topology have occurred in our estimates, these changes should not substantially impact the qualitative conclusions of further analyses (e.g., sliding window analysis, SWA) because a majority of these later estimates are averaged over many branches of the tree, and the dynamics we concentrate on are quite dramatic and are likely to be obvious and qualitatively similar even with slight changes in the topology estimate.

### Nucleotide frequencies and control region functionality

In *A. piscivorus *and *P. slowinskii *mt genomes, as in other vertebrates [5], nucleotides A and C are favored on the light strand, particularly at 3^rd ^codon positions. This bias is probably related to elevated rates of deamination mutations on the heavy strand incurred during replication (see Background), and there is considerable variation in nucleotide content among individual mt genomes (see Additional file 2). Variation in snakes, even at 3^rd ^codon positions, is not exceptional compared to other groups, and there is no clear snake-specific nucleotide bias evident (see Additional file 2) or strong branch-specific, or gene-specific nucleotide bias shifts across squamate mt genomes that would explain our findings of dramatic branch-specific and gene-specific rate dynamics.

Due to the simple linear relationship in most vertebrate mt genomes between C/T ratios and *T*_*AMS *_predicted based on the location of the (functional) control region, it is of interest to determine whether there has been any clear genetic effect of the duplicated control region in alethinophidians. Exclusive use of one control region or the other would be most strongly observable in ND1 (the only protein-coding gene located between the two control regions in alethinophidian snake mt genomes) because it is the only protein-coding gene that would spend a substantially different amount of time in the asymmetric mutagenic state (*T*_*AMS *_see Additional file 2) depending on which control region is functional. Since the nucleotide sequence of duplicate control regions is nearly identical within each genome, however, it is also reasonable to consider the possibility that both control regions are functional.

To test these predictions about CR2 functionality, we applied our MCMC analysis [3] to fit alternative models of exclusive CR1 or CR2 usage, or mixed control region effect (Table 1). The Akaike weights for the alternative individual models provide a measure of the degree to which a control region is exclusively functional, while the weight parameter in the mixed model represents the time-averaged effect of mixed control region usage on the C/T ratios. There is evidence for at least mixed CR2 usage in all but one species (*Cylindrophis ruffus*). The evidence is good for exclusive or nearly exclusive CR2 functionality in two species (*Acrochordus granulatus *and *Python regius*), and for a strong CR2 preference in *A. piscivorus*. The patterns appear to be lineage-specific and evolutionarily labile (i.e., strong preferences for a particular control region are widely dispersed on the tree), which may indicate rapid evolution of the strength of the replication-associated substitution gradient (as suggested in primates [3]) or rapid evolution of differential usage of the two control regions. Species with ambiguous control region preferences may have mixed usage, may not have a strong enough gradient to differentiate, or may have previously switched usage and thus have not reached mutational equilibrium.

**Table 1:** Results of mitochondrial genome replication model analyses

	**Individual model**		**Mixed model**	
	
**Species**	OHCR1 MathType@MTEF@5@5@+=feaafiart1ev1aaatCvAUfKttLearuWrP9MDH5MBPbIqV92AaeXatLxBI9gBaebbnrfifHhDYfgasaacH8akY=wiFfYdH8Gipec8Eeeu0xXdbba9frFj0=OqFfea0dXdd9vqai=hGuQ8kuc9pgc9s8qqaq=dirpe0xb9q8qiLsFr0=vr0=vr0dc8meaabaqaciaacaGaaeqabaqabeGadaaakeaacqWGpbWtdaqhaaWcbaGaemisaGeabaGaem4qamKaemOuaiLaeGymaedaaaaa@3245@	OHCR2 MathType@MTEF@5@5@+=feaafiart1ev1aaatCvAUfKttLearuWrP9MDH5MBPbIqV92AaeXatLxBI9gBaebbnrfifHhDYfgasaacH8akY=wiFfYdH8Gipec8Eeeu0xXdbba9frFj0=OqFfea0dXdd9vqai=hGuQ8kuc9pgc9s8qqaq=dirpe0xb9q8qiLsFr0=vr0=vr0dc8meaabaqaciaacaGaaeqabaqabeGadaaakeaacqWGpbWtdaqhaaWcbaGaemisaGeabaGaem4qamKaemOuaiLaeGOmaidaaaaa@3247@	OHCR1+OHCR2 MathType@MTEF@5@5@+=feaafiart1ev1aaatCvAUfKttLearuWrP9MDH5MBPbIqV92AaeXatLxBI9gBaebbnrfifHhDYfgasaacH8akY=wiFfYdH8Gipec8Eeeu0xXdbba9frFj0=OqFfea0dXdd9vqai=hGuQ8kuc9pgc9s8qqaq=dirpe0xb9q8qiLsFr0=vr0=vr0dc8meaabaqaciaacaGaaeqabaqabeGadaaakeaacqWGpbWtdaqhaaWcbaGaemisaGeabaGaem4qamKaemOuaiLaeGymaedaaOGaey4kaSIaem4ta80aa0baaSqaaiabdIeaibqaaiabdoeadjabdkfasjabikdaYaaaaaa@38CC@	***% ***OHCR2 MathType@MTEF@5@5@+=feaafiart1ev1aaatCvAUfKttLearuWrP9MDH5MBPbIqV92AaeXatLxBI9gBaebbnrfifHhDYfgasaacH8akY=wiFfYdH8Gipec8Eeeu0xXdbba9frFj0=OqFfea0dXdd9vqai=hGuQ8kuc9pgc9s8qqaq=dirpe0xb9q8qiLsFr0=vr0=vr0dc8meaabaqaciaacaGaaeqabaqabeGadaaakeaacqWGpbWtdaqhaaWcbaGaemisaGeabaGaem4qamKaemOuaiLaeGOmaidaaaaa@3247@
***Agkistrodon piscivorus***	1179.2 (18%)	1178.0 (60%)	1179.0 (22%)	99%
***Pantherophis slowinskii***	1164.6 (29%)	1164.1 (47%)	1164.8 (24%)	54%
***Dinodon semicarinatus***	1167.1 (21%)	1166.2 (57%)	1167.1 (22%)	78%
***Ovophis okinavensis***	1252.7 (38%)	1252.6 (45%)	1253.5 (17%)	59%
***Boa constrictor***	854.5 (29%)	853.9 (50%)	854.8 (21%)	64%
***Acrochordus granulatus***	1245.0 (2%)	1241.5 (72%)	1242.5 (26%)	100%
***Xenopeltis unicolor***	1159.4 (31%)	1159.0 (45%)	1159.6 (24%)	50%
***Python regius***	1133.0 (1%)	1128.9 (72%)	1130.0 (26%)	100%
***Cylindrophis ruffus***	1129.8 (70%)	1132.6 (4%)	1130.8 (26%)	< 1%

Negative log likelihood values and Akaike weights (in parentheses) for individual origin of replication models and the mixed model, along with the most likely CR2 preference parameter in the mixed model, for alethinophidian snakes.

### Gene length and stability of truncated tRNAs in snakes

In snakes, all mt protein-coding genes (except COX1), ribosomal RNAs, tRNAs, and individual CRs are shorter than their counterparts in most lizards and most other vertebrates (see Additional file 3). An exception to this is *Sphenodon punctatus*, for which the control region, ATP8 (ATP synthase subunit 8) and the 12s rRNA are all shorter than in snakes. With the increased sampling in this study, it appears that while the tRNAs and proteins became shorter prior to the divergence of all snakes, the tRNAs became shorter still within the Colubroidea (Figures 4 and Additional file 3). Additionally, the rRNAs did not become shorter in *L. dulcis *or the Acro-Heno clade, but are dramatically shorter in the Colubroidea (Figures 4 and Additional file 3).

The shorter length of tRNAs in snakes results mainly from a truncated T-arm in the secondary structure (see also [8,9]). In some tRNAs, the D-arm is also shorter, but to a lesser extent than the T-arms. Although short tRNAs are typically less stable than long ones, there is only a minor effect of sequence length on secondary structure stability (ΔG) in snake tRNAs. The cloverleaf structures of most snake tRNAs are slightly less stable than their lizard counterparts (see Additional file 2), but two tRNAs (tRNA^Ile^, tRNA^Met^) are actually more structurally stable in snakes than in other squamates with longer tRNAs.

### Spatio-temporal substitution rate dynamics across mt genes and genomic regions

**Figure 5:**
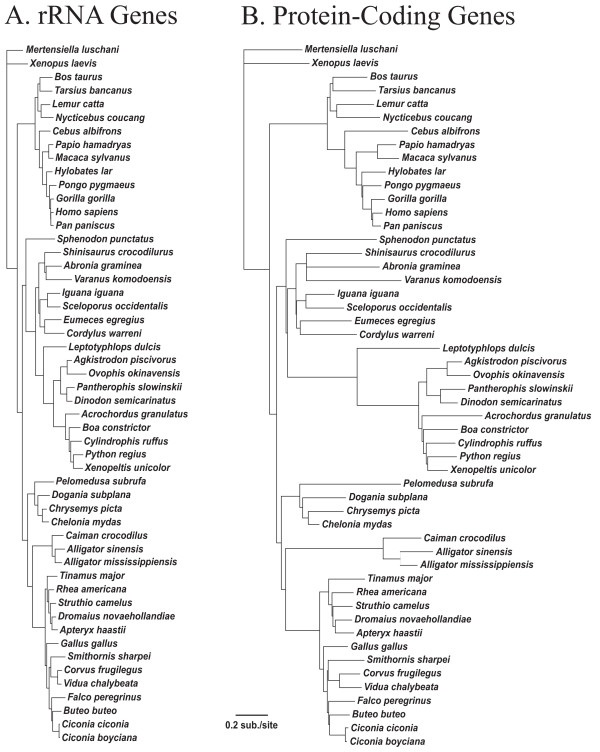
**Phylograms based on the relative branch lengths for rRNA and protein-coding genes**. Branch lengths were estimated on the topology of the ML phylogeny (Figure 3). Branch lengths on this constrained topology were estimated using all rRNA genes (A) or all protein-coding genes (B). The substitution rate scale is the same in both trees.

To assess the difference in substitution rates among genes, we fixed the topology (to that in Figure 3) and calculated branch lengths based on rRNAs and on all protein-coding genes (Figure 5). For the rRNAs, most other major amniote groups have experienced similar amounts of total evolution from their common ancestor with the amphibians (i.e., the branch lengths from the root to the terminals are similar), whereas the snake lineages stand out as unusual in their apparently accelerated evolution (i.e., their exceptionally long root to tip branch lengths; Figure 5A). For protein-coding genes there is much more variation across lineages (Figure 5B), although the snake clade has the longest branches of any tetrapod group. Certain snake branches (e.g., the branch leading to all snakes and to the Alethinophidia) are disproportionately long compared to branch lengths based on rRNAs (Figure 5). To evaluate this further, branch lengths were calculated for different genes and gene clusters and there was considerable variation among genes with respect to relative branch lengths in early snake lineages (data not shown).

**Figure 6:**
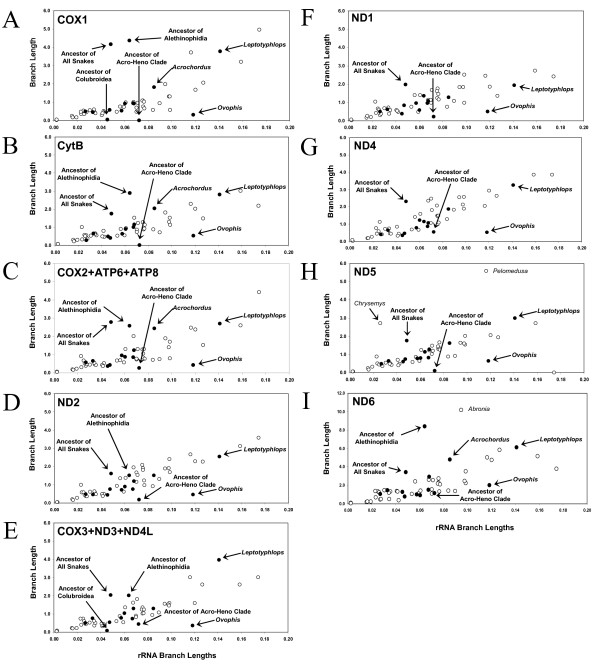
**Plot of branch lengths obtained from rRNA versus various genes and gene clusters**. Branches leading to the most recent common ancestor of a group are labeled e.g. "Ancestor of All Snakes". Snake branches are indicated with filled circles, and non-snake tetrapod branches are indicated with an unfilled circle. The locations of selected snake branches are labeled (in bold) with arrows. Outlying non-snake branches are indicated and labeled in normal type. Genes and gene clusters shown are (A) COX1, (B) CytB, (C) COX2 + ATP6 + ATP8, (D) ND2, and (E) COX3 + ND3 + ND4L, (F) ND1, (G) ND4, (H) ND5, (I) ND6.

To qualitatively elucidate the spatio-temporal dynamics in rates of substitution between gene regions that occur across branches, we plotted the branch lengths derived from rRNAs (which appear to have had little or no acceleration; e.g., Figure 5A) versus the branch lengths of various genes and gene clusters (Figure 6). All gene pairs generally appear to have highly correlated branch lengths (Figure 6), but some branches are outside the main distribution. Two branches consistently below the main distribution in most comparisons are the terminal branch leading to *O. okinavensis *and the branch leading to the Acro-Heno clade (Figure 6); these two branches are also disproportionally longer in the rRNA trees than in the protein trees (Figure 5). These branches appear to have experienced acceleration of rRNA genes well beyond the mild accelerated evolution of rRNA that occurred along the lineages leading to all snakes and to the Alethinophidia.

**Figure 7:**
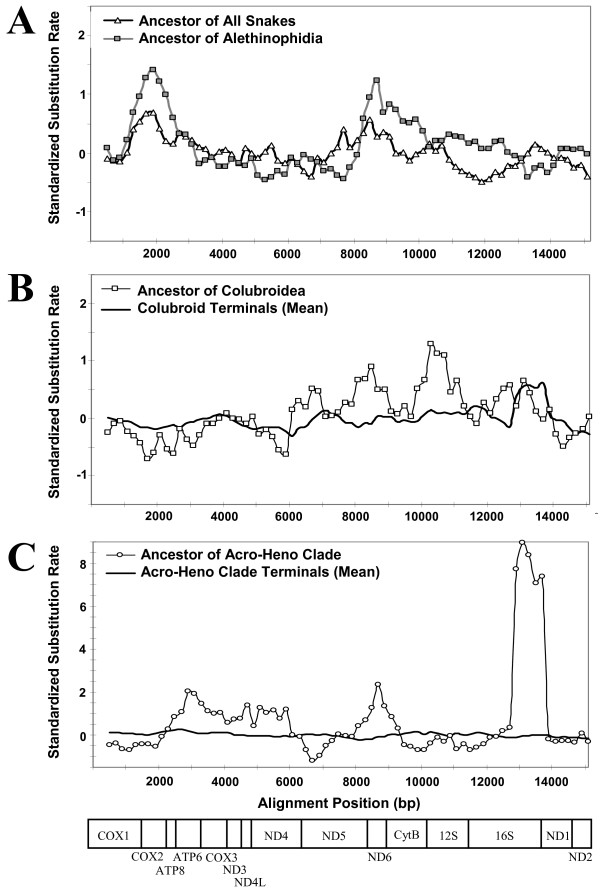
**Standardized substitution rates across the mitochondrial genome for selected branches or clusters**. For each 1000 bp window applied to a set of branches, standardized substitution rates were obtained by first dividing by the median window value for that branch, and then subtracting this value from the average across all non-snake branches. This helps to visualize regions of the genome that are evolving at slower or faster rates, with the average tetrapod relative rate being zero. Branches or branch sets shown are (A) the branch leading to the most recent common ancestor of all snakes and of the Alethinophidia; (B) the branch leading to the most recent common ancestor of the Colubroidea and the sum of all colubroid terminal branches; and (C) the branch leading to the most recent common ancestor of the Acro-Heno clade and the sum of all Acro-Heno clade terminal branches.

To further evaluate the variation in spatio-temporal dynamics of relative rates of substitution across the mt genome, we used sliding window analyses of branch-specific and group-specific patterns of relative substitution rates. Only one of these comparisons, that of the Acro-Heno clade terminal branches, shows little variation of standardized relative substitution rates across the genome (Figure 7C). This suggests that the distribution of substitutions across the mt genome of terminal lineages within the Acro-Heno clade is nearly identical to the distribution across the mt genome of other tetrapods, and that these terminal snake lineages are not undergoing region or gene-specific selection. The plots for terminal colubroid branches are also fairly flat except for the downstream half of the 16s rRNA (Figure 7B), which may be entirely attributable to acceleration of the 16s rRNA in *O. okinavensis*, as discussed earlier.

Cross-referencing results from Figures 5, 6, 7, we can summarize the apparent nucleotide evolutionary rate dynamics in snake mt genomes as follows (see also Figure 4). The branch leading to all extant snakes appears to have experienced accelerated evolution in the region starting near the end of COX1 through COX2, ATP8, and somewhat into ATP6, and also in the region including the end of ND5, ND6, and CytB (and a rise in ND1). The COX1, COX2, ATP8, and ND6 accelerations increased and were stronger in the branch leading to Alethinophidia, while the ND5 acceleration decreased, and a notable acceleration of CytB also occurred. In the branch leading to the Colubroidea, only the ND6 acceleration continued, but new rate peaks arose in ND5, 12s rRNA, and the first part of the 16s rRNA, followed by a strong dropoff in all gene-specific acceleration in terminal colubroid lineages, except in the end of 16s rRNA in *O. okinavensis*. In the branch leading to the Acro-Heno clade, the accelerated rates of evolution (in COX1, COX2, ATP8, and ND5 genes) observed along the branch leading to the alethinophidians diminished (except for ND6 as in the Colubroidea), but new rate peaks arose in ATP6, COX3, ND3, ND4L, and the latter half of the 16s rRNA. These punctuated gene-specific accelerations were followed by the complete elimination of all gene-specific signals of atypical relative rate in terminal lineages within this Acro-Heno clade. We find no evidence for a constant accelerated rate of snake mt genome evolution. Instead, our analyses of rates and patterns of substitution underscore both the spatial (gene-specific) and temporal (branch-specific) nature of molecular evolutionary relative rate dynamics in snake mt genomes.

## Discussion

The three new complete snake mt genomes presented here, together with previously existing vertebrate mt genomes, provide a preliminary perspective on a complex history of potentially adaptive mt genomic change in snakes. Unusual changes in gene size and nucleotide substitution rates are associated with changes in mt genomic architecture (Figure 4). Nevertheless, the changes in substitution dynamics cannot be directly explained by the changes in mt genome architecture. Snake mt genome evolution is most consistent with some type of broad selective pressure on the efficiency and function of oxidative metabolism in snakes early in their evolutionary history.

In mt genomes (particularly in vertebrates), the processes of replication and transcription are not entirely functionally independent, and genome structural organization plays a prominent role in both processes. The CR acts as the origin of heavy strand replication, in addition to its role as the promoter for both heavy and light strand transcription [18]. Genome replication also depends on the processing of light strand transcripts to produce short primers required for heavy strand initiation of genome replication (originating from the CR [19]). The regular distribution of the tRNA genes throughout the mt genome is functionally significant, and these play an important role in RNA processing of polycistrons to yield mature RNAs, transcription initiation and termination, as well as initiation of light strand replication [18]. Collectively, many functional ramifications are linked tightly to mt genome architecture in vertebrates.

Mitochondrial genome size reduction due to gene shortening in alethinophidians is more than offset by the retention of their duplicate control regions. If size reduction is caused by selective pressure, the long term retention of dual CRs suggests that having both copies provide some selective advantage. Although the duplicate control region appears to function in heavy strand replication in at least some snakes, there is considerable variation in CR usage across snake lineages (Table 1). Thus, if the duplication has been maintained by selection, control of replication may not be the singular or primary selective driving force.

The possession of two functional control regions in most snake mt genomes might be advantageous by increasing the rate at which genome replication proceeds, and/or increasing the overall number of genome copies per mitochondrion. Since the dual CRs essentially flank the rRNA genes, they (along with adjacent tRNAs) could also plausibly function to independently control rates of protein-coding and rRNA gene transcription. Across snake species, variation in the tRNAs flanking the CRs includes the translocation of tRNA^Leu ^(3' of CR2) and the duplication/translocation/truncation of tRNA^Pro^. In vertebrates, tRNA^Leu ^has been shown to decouple rates of rRNA and mRNA transcription by acting as a terminator of ~95% of heavy strand transcripts (leading to ~20-fold higher rRNA vs. mRNA levels; [18]). Considering the ectothermy of snakes, transcriptional decoupling via independent control regions could provide a more direct means of countering thermodynamic depression of enzymatic rates at low temperatures.

Independent CR duplications have also been identified in eels [20], frogs [21], birds [22,23], and lizards [24,25]. Our results (and additional unpublished data) suggest that the dramatic shifts in rates and patterns of molecular evolution in snakes represent a unique phenomenon that we do not expect to be necessarily associated with CR duplication, but rather more likely associated with selection for mitochondrial function. Nevertheless, these independent duplications may be useful to test the consequences of duplication on mutational processes.

### Concerted evolution in and around the duplicate control regions

The two control regions clearly undergo concerted evolution to maintain reciprocal homogeneity between control regions within a genome [7-9], presumably through gene conversion. Interestingly, an apparently nonfunctional partial (or pseudo) proline tRNA (Ψ-tRNA^Pro^) in colubrid mt genomes also appears to be maintained by concerted evolution (Figure 1). The gene conversion process that homogenizes the control region may also occasionally pick up extra DNA, making tRNA^Pro^, or part of it, prone to duplication at this location. The existence of a duplicate tRNA^Phe ^between CR2 and tRNA^Leu ^in the viperid *O. okinavensis *[7] suggests that frequent gene duplication adjacent to the CRs may occur (these two tRNA^Phe ^differ by only 3 of 64 bp; implying either concerted evolution or recent duplication). The concerted evolution of these tRNAs could be explained by a tendency for gene conversion events involving the duplicate control regions to extend into the homologous flanking tRNA regions.

Another point of interest concerning gene conversion that arises from this study is a preliminary indication of differential evolutionary processes operating on the CRs within versus between species. Vertebrate mitochondrial control regions typically evolve very rapidly, and this is the case in a comparison of the two viperid species (*O. okinavensis *and *A. piscivorus*) in which CRs from these species are (on average) approximately as divergent as the fastest evolving positions within the mt genome, third codon positions (Figure 2B). In contrast, the two *A. pisvicorus *genomes, *Api1 *and *Api2*, have surprisingly similar CRs between individuals (Figure 2A; Additional file 2), comparable to the similarity between rRNA genes, among the slowest evolving regions in the mt genome. A previous study on viperid snakes also showed slow within-species CR evolutionary rates [26], and other studies have demonstrated particularly slow intra-species rates and differential rates of CR evolution operating within versus between species in birds [27] and fish [28].

In this study we have found a great deal of rate heterogeneity among genes, so it is certainly possible that the normally unconserved control regions have become suddenly critical and conserved in *A. piscivorus*. Alternatively, it is plausible that the complex (and poorly understood) process of gene conversion of CRs within a genome may also alter rates of CR evolution within species through a yet unknown process of gene conversion that may involve intragenomic (or even intergenomic) recombination.

### Comparative rates of molecular evolution

Previous studies have suggested that snake mt genomes have an accelerated rate of evolution [7,8]. Our results suggest this general conclusion is an oversimplification of a much more complex scenario, and that rates of snake mt genome evolution incorporate broad temporal (branch-specific) and spatial (gene and gene region-specific) dynamics. Branches early in snake evolution appear to be associated with dramatically elevated evolutionary rates and extreme relative rate dynamics across the mt genome (Figure 4). In contrast, terminal branches appear to have patterns of mt genome evolution that are strikingly similar to other (non-snake) vertebrates.

In support of a hypothesis involving selection for overall oxidative metabolic function, the accelerated rates of molecular evolution in snakes appear to depend greatly on gene function, with most ND subunits accelerating only slightly and occasionally, while COX, ATP, CytB, and rRNA evolutionary accelerations are dramatic and punctuated. The roles of these proteins (and the mitochondria in general) in energetics via oxidative phosphorylation are well known, and it may be that a single causative agent accompanying the diversification of snakes that dramatically altered metabolic demand, or led to a fluctuation in metabolic demand, was responsible for large-scale changes in selective pressure on these proteins.

## Conclusion

Snake mitochondrial genomes present a rare opportunity to investigate the evolutionary interactions and ramifications that link genome architecture, molecular evolution, and multi-level molecular function. Available evidence points to selective pressures acting at many hierarchical levels within snake mt genomes, and at different times during snake evolution, leading to diverse, dramatic, and broad-scale changes in the genome. Interestingly, some consequences of this adaptive shift appear to have diminished over time (e.g., accelerated evolutionary rates of COX and other genes), whereas others appear to continue in extant snakes (i.e., the effects of control region duplication on mutation gradients, replication, and potentially transcription, and remnant functional consequences of short and highly substituted genes). Although the precise causes are unknown, this outstanding example of an apparent punctuated adaptive shift involving multiple aspects of genome architecture evolution provides an important comparative tool for the study of vertebrate mt genome evolution.

## Methods

### Sampling, sequencing and annotation

DNA was extracted from vouchered specimens available at the Louisiana State University Museum of Natural Science (LSUMZ) and the University of Central Florida (CLP). The *Agkistrodon piscivorus *(cottonmouth or water moccasin; Viperidae) specimens were from Louisiana, USA (LSUMZ-17943) and from Florida, USA (CLP-73). We refer to these as *Api1 *(Louisiana specimen) and *Api2 *(Florida specimen). The *Pantherophis slowinskii *(corn snake; Colubridae) specimen was from Louisiana, USA (LSUMZ- H-2036). The genus *Pantherophis *[29] was recently erected to contain a clade of species formerly allocated to *Elaphe*. The species *P. slowinskii *was formerly considered *Pantherophis *(*Elaphe*)*guttatus*, and was recently recognized as a distinct species [30]. Details of molecular laboratory methods (e.g., PCR, cloning, sequencing), genome annotation [31], and accession numbers are provided in Additional files (see Additional files 2 and 4).

### Phylogenetic and sliding-window analyses

In addition to the three new snake mt genome sequences, the sequence dataset used included all eight snake mt genomes available at the time of the study, and 42 additional taxa for comparative purposes, including heavy sampling of birds, mammals (mostly primates), and lizards (species scientific names and access numbers are given in Additional file 2). Sequences of protein-coding and rRNA genes were aligned using ClustalX [32], followed by manual adjustment. Protein-coding genes were first aligned at the amino acid level, and then the nucleotide sequences were aligned according to the corresponding amino acid alignment. The alignment of rRNAs contained a small number of sites (corresponding to the loop-forming structures of the rRNAs) with somewhat ambiguous alignments only among major tetrapod lineages. Since we wanted to compare estimates of mitochondrial gene evolutionary rates and patterns, we chose not to exclude any sites of the alignment. This was also justified by preliminary phylogenetic estimates that suggested the incorporation of these few potentially ambiguous sites did not affect phylogenetic results. The main phylogeny presented here was inferred using the concatenated nucleotide sequence of all 13 protein-coding and two rRNA genes by maximum-likelihood (ML) analysis in PAUP 4.0 beta10 [33]. This analysis used the GTR + Γ + I model of evolution, the best-fit model under all criteria in ModelTest [34].

Support for this topology was evaluated in two ways: (1) based on 1000 NJ bootstraps (in PAUP) with ML distances calculated under the same model as above, but with down-weighted synonymous sites to avoid saturation problems (rRNAs relative weight = 5 and 1^st^, 2^nd^, and 3^rd ^codon positions relative weights = 4, 5, and 1) and (2) based on Bayesian posterior probability support estimated by conducting two simultaneous independent MCMC runs conducted for 10^6 ^generations (with the first 400,000 generations of each run discarded as burn-in) using a GTR + Γ + I model of evolution (in MrBayes 3.1 [17]). The burnin period was determined by visual assessment of stationarity and convergence of likelihood values between the chains. To analyze nucleotide substitution rate variation in different lineages and different genes, branch length estimates were separately calculated under the GTR + Γ + I model for different genes (COX1, ND1, ND2, ND4, ND5, CytB) and gene clusters (COX2 + ATP8 + ATP6, and COX3 + ND3 + ND4L; each comprising groups of individually short genes adjacent along the mt genome) using the ML topology and PAML [35]. To further analyze fluctuations in nucleotide substitution rates, we conducted sliding window analyses (SWA) on the phylogenetic dataset. The program Hyphy [36] was used to estimate branch lengths (estimated numbers of substitutions) for 1000 bp windows. SWA was conducted using the GTR model with global parameter estimation and topological relationships specified based on the ML tree estimate, with a window slide of 200 bp. Based on preliminary trials, the size of the window and slide length were chosen to minimize noise observed with shorter windows, but to allow differentiation of patterns in different regions. To compare patterns of substitution across the mitochondrial genome for select branches or groups of branches, we first divided substitution estimates for each window by the median substitution rate across all windows. Since branch lengths are estimates of *δ*_*b*_*t*_*b *_(the branch-specific substitution rate times divergence time) this procedure estimates a ratio of substitution rates, δbw/δbξ
 MathType@MTEF@5@5@+=feaafiart1ev1aaatCvAUfKttLearuWrP9MDH5MBPbIqV92AaeXatLxBI9gBaebbnrfifHhDYfgasaacH8akY=wiFfYdH8Gipec8Eeeu0xXdbba9frFj0=OqFfea0dXdd9vqai=hGuQ8kuc9pgc9s8qqaq=dirpe0xb9q8qiLsFr0=vr0=vr0dc8meaabaqaciaacaGaaeqabaqabeGadaaakeaaiiGacqWF0oazdaqhaaWcbaGaemOyaigabaGaem4DaChaaOGaei4la8Iae8hTdq2aa0baaSqaaiabdkgaIbqaaiab=57a4baaaaa@3711@, where δbw
 MathType@MTEF@5@5@+=feaafiart1ev1aaatCvAUfKttLearuWrP9MDH5MBPbIqV92AaeXatLxBI9gBaebbnrfifHhDYfgasaacH8akY=wiFfYdH8Gipec8Eeeu0xXdbba9frFj0=OqFfea0dXdd9vqai=hGuQ8kuc9pgc9s8qqaq=dirpe0xb9q8qiLsFr0=vr0=vr0dc8meaabaqaciaacaGaaeqabaqabeGadaaakeaaiiGacqWF0oazdaqhaaWcbaGaemOyaigabaGaem4DaChaaaaa@3149@ is the branch- and window-specific substitution rate, and δbξ
 MathType@MTEF@5@5@+=feaafiart1ev1aaatCvAUfKttLearuWrP9MDH5MBPbIqV92AaeXatLxBI9gBaebbnrfifHhDYfgasaacH8akY=wiFfYdH8Gipec8Eeeu0xXdbba9frFj0=OqFfea0dXdd9vqai=hGuQ8kuc9pgc9s8qqaq=dirpe0xb9q8qiLsFr0=vr0=vr0dc8meaabaqaciaacaGaaeqabaqabeGadaaakeaaiiGacqWF0oazdaqhaaWcbaGaemOyaigabaGae8NVdGhaaaaa@3190@ is the branch-specific substitution rate in the median window. To evaluate whether the windows had relative rates that were slower or faster than expected, we took the substitution rate ratio from the set of all branches in the non-snakes (NS) as a standard. This was then subtracted from the branch-specific ratio to obtain a "standardized substitution rate",δbw/δbξ−δNSw/δNSξ
 MathType@MTEF@5@5@+=feaafiart1ev1aaatCvAUfKttLearuWrP9MDH5MBPbIqV92AaeXatLxBI9gBaebbnrfifHhDYfgasaacH8akY=wiFfYdH8Gipec8Eeeu0xXdbba9frFj0=OqFfea0dXdd9vqai=hGuQ8kuc9pgc9s8qqaq=dirpe0xb9q8qiLsFr0=vr0=vr0dc8meaabaqaciaacaGaaeqabaqabeGadaaakeaaiiGacqWF0oazdaqhaaWcbaGaemOyaigabaGaem4DaChaaOGaei4la8Iae8hTdq2aa0baaSqaaiabdkgaIbqaaiab=57a4baakiabgkHiTiab=r7aKnaaDaaaleaacqWGobGtcqWGtbWuaeaacqWG3bWDaaGccqGGVaWlcqWF0oazdaqhaaWcbaGaemOta4Kaem4uamfabaGae8NVdGhaaaaa@446F@. When relative rates of substitution are distributed similarly across the mt genome, in comparison with NS, this standardized rate comparison approaches zero.

### tRNA structure

The secondary structures of squamate tRNAs were determined under the guidance of the mammalian tRNA cloverleaf structures [37] and the tRNAscan program [38], and then used to modify tRNA alignments by hand (tRNA^Ser ^[AGY] was not included in these analyses because it does not form a cloverleaf structure). To determine the relative stabilities of the tRNA secondary structures, we calculated the energy (Δ*G*) of the cloverleaf structure using the Vienna Package version 1.4 [39].

### Analysis of control region functionality

The calculation of *T*_*AMS *_differs depending on whether CR1 or CR2 is functional, but only for the genes that are positioned between the two control regions, the two rRNAs and ND1 (see Additional file 2). Based on previous work, the light strand C/T ratio at synonymous two-fold and fourfold redundant 3^rd ^codon positions is expected to increase linearly with *T*_*AMS*_, so we used this prediction to determine whether there was any evidence for activity of CR1 or CR2 in initiating heavy strand replication. We implemented a slightly modified version of the MCMC approach in [3] to estimate the most likely slope and intercept of the C/T ratio gradient depending on the calculated *T*_*AMS *_at every site. We applied these calculations using *T*_*AMS *_from CR1 and CR2, and also separately calculated the slope and intercept for the most likely weighted average *T*_*AMS *_for the two control regions. Other than the addition of the weighting parameter, all details of the Markov chain were as in [3]. Relative support for alternative hypotheses was determined using Akaike Information Criterion (AIC) and Akaike weights [40,41].

## Abbreviations

rRNA, tRNA : ribosomal RNA, transfer RNA

mt: mitochondrial

O_H_: origin of heavy strand replication

CR, CR1, CR2: control region, control region 1, control region 2

O_L_: origin of light strand replication

ND#: NADH dehydrogenase subunit #

COX#: Cytochrome C oxidase subunit #

D_ssH_: Duration of time spent single-stranded by the heavy strand during replication

T_AMS_: Time spent in an asymmetric mutagenic state during replication

C, T, A, G: cytosine, thymine, adenine, guanine

CytB: cytochrome b

ATP#: ATP synthase subunit #

Ile, Met, Pro, Thr, Leu, Phe, Ser: isoleucine, methionine, proline, threonine, leucine, phenylalanine, serine

SWA: sliding window analysis

MYA: million years ago

LSUMZ: Louisiana State University Museum of Natural Science specimen tag

CLP: University of Central Florida specimen tag

Api1, Api2: *Agkistrodon piscivorus *specimen #

NS: non-snakes

## Authors' contributions

ZJJ co-wrote the manuscript, performed much of the data analysis, and participated in sequencing of *P. slowinskii *and Api1. TAC co-wrote the manuscript, performed much of the data analysis, and participated in sequencing of Api2. CCA helped manage the project and assisted in writing and editing the manuscript. FTB performed the primary sequencing of *P. slowinskii *and Api1 and edited the manuscript. MDH performed the preliminary sequencing of Api2 and edited the manuscript. JAM contributed to the design and conception of the project and edited the manuscript. CLP supervised the sequencing of Api2 and edited the manuscript. DDP co-wrote the manuscript, designed and conceived the project, and supervised the sequencing of *P. slowinskii *and Api1 and the analysis of the data.

## Supplementary Material

Additional file 1 Mitochondrial genome replication and substitution gradients background. Click here for file

Additional file 2 Laboratory and genome annotation methods. Click here for file

Additional file 3 Comparison of gene lengths in snakes and other squamates. Click here for file

Additional file 4 All supplementary tables. Click here for file
