# Supplementary material for: Comparative mitochondrial genomics of snakes: extraordinary substitution rate dynamics and functionality of the duplicate control region
Source: BMC Evol Biol. 2007 Jul 26;7:123. doi: 10.1186/1471-2148-7-123 (PMC1950710; doi:10.1186/1471-2148-7-123)
Supplement: Additional file 1 — Mitochondrial genome replication and substitution gradients background. [file 1471-2148-7-123-S1.pdf]

Additional File 2 (Supplementary Background)

Supplementary Background. Mitochondrial Replication and Transition Biases.

The mt genome has long been believed to replicate asymmetrically [1], which strongly contributes to the substantial difference in mutation rates and nucleotide composition biases between strands [2-6]. During replication under the classical model, the synthesis of the nascent heavy strand initiates at the origin of heavy strand replication (O_H), within the control region (CR). This has been reviewed extensively elsewhere [2, 7], but in brief, after two thirds of the nascent heavy strand is synthesized, the synthesis of the nascent light strand starts at the origin of light strand replication (O_L), a short secondary-structure-forming segment located within the tRNA cluster (the WANCY region) between the NADH dehydrogenase subunit 2 (ND2) and Cytochrome C oxidase subunit 1 (COX1) genes. The strand-asymmetric replication mechanism has been thought to expose different regions of the parental heavy strand to varying amounts of time in the single-stranded state during replication (D_{ssH}; [6]), depending on the distances of the regions from the O_H and O_L. Variation in this strand-asymmetric replication process appears to have contributed substantially to variation in substitution rates among genes [2, 7, 8].

Controversy has arisen recently over the classical mt genome replication mechanism, mostly concerning the asymmetry of the process, the role of the putative origin of light strand replication, and whether the replicating DNA spends substantial amounts of time single-stranded [9-11]. Although newly proposed models of replication that embody these concerns are directly at odds with the genetic data, one of us has hypothesized

(Pollock, *in review*) that most of the biochemical and genetic data are compatible with a reconciled model of mt genome replication, which retains most critical features of the classical model except for single strandedness. Regardless of the final reconciliation, here we take a neutral position on the biochemical issue of single-strandedness by referring to the time that a gene or nucleotide is predicted to spend in an asymmetric mutagenic state (T_{AMS}), rather than the predicted duration of time that the heavy strand spends single-stranded (D_{SSH}); the calculation of T_{AMS} is, however, identical to that for D_{SSH} [6, 7, 12].

Cytosine →Uracil deaminations are common in single-stranded DNA, while Adenine → Hypoxanthine deaminations are less common [13, 14]. These two deaminations lead to mutations (Cytosine→Thymine and Adenine→Guanine, or C→T and A→G) that appear to account for most of the asymmetry in synonymous substitutions found in vertebrate mt genome [7, 8, 12, 15-19]. C→T and A→G mutations on the heavy strand during replication apparently lead respectively to G→A and T→C substitutions (and G and T deficiencies) on the light strand. Most protein-coding genes (all but ND6) use the heavy strand as a template; thus, the mutation biases observed in the light strand parallel the biases in most protein-coding gene transcripts. Faith and Pollock [7] found that, in vertebrates, T→C light strand substitutions at four-fold and two-fold redundant 3rd codon positions increase linearly with increasing T_{AMS}. In contrast, G→A light strand substitutions increase rapidly but quickly reach a maximal level. Consequently, T→C substitutions and the resultant C/T nucleotide frequency gradient are good predictors of T_{AMS}.

References

1. Clayton DA: **Replication of animal mitochondrial DNA**. *Cell* 1982, **28**(4):693-705.
2. Bielawski JP, Gold JR: **Mutation patterns of mitochondrial H- and L-Strand DNA in closely related cyprinid fishes**. *Genetics* 2002, **161**(4):1589-1597.
3. Jermiin LS, Graur D, Crozier RH: **Evidence from analyses of intergenic regions for strand-specific directional mutation pressure in metazoan mitochondrial DNA**. *Mol Biol Evol* 1995, **12**(4):558-563.
4. Perna NT, Kocher TD: **Patterns of nucleotide composition at fourfold degenerate sites of animal mitochondrial genomes**. *J Mol Evol* 1995, **41**(3):353-358.
5. Perna NT, Kocher TD: **Unequal base frequencies and estimation of substitution rates**. *Mol Biol Evol* 1995, **12**(2):359-361.
6. Tanaka M, Ozawa T: **Strand asymmetry in human mitochondrial-DNA mutations**. *Genomics* 1994, **22**(2):327-335.
7. Faith JJ, Pollock DD: **Likelihood analysis of asymmetrical mutation bias gradients in vertebrate mitochondrial genomes**. *Genetics* 2003, **165**(2):735-745.
8. Raina SZ, Faith JJ, Disotell TR, Seligmann H, Stewart CB, Pollock DD: **Evolution of base-substitution gradients in primate mitochondrial genomes**. *Genome Res* 2005, **15**(5):665-673.
9. Reyes A, Yang MY, Bowmaker M, Holt IJ: **Bidirectional replication initiates at sites throughout the mitochondrial genome of birds**. *J Biol Chem* 2005, **280**(5):3242-3250.
10. Yang MY, Bowmaker M, Reyes A, Vergani L, Angeli P, Gringeri E, Jacobs HT, Holt IJ: **Biased incorporation of ribonucleotides on the mitochondrial L-strand accounts for apparent strand-asymmetric DNA replication**. *Cell* 2002, **111**(4):495-505.
11. Yasukawa T, Yang MY, Jacobs HT, Holt IJ: **A bidirectional origin of replication maps to the major noncoding region of human mitochondrial DNA**. *Mol Cell* 2005, **18**(6):651-662.
12. Reyes A, Gissi C, Pesole G, Saccone C: **Asymmetrical directional mutation pressure in the mitochondrial genome of mammals**. *Mol Biol Evol* 1998, **15**(8):957-966.
13. Frederico LA, Kunkel TA, Shaw BR: **A sensitive genetic assay for the detection of cytosine deamination - determination of rate constants and the activation energy**. *Biochemistry (Mosc)* 1990, **29**(10):2532-2537.
14. Impellizzeri KJ, Anderson B, Burgers PMJ: **The spectrum of spontaneous mutations in a *Saccharomyces cerevisiae* Uracil-DNA-Glycosylase mutant limits the function of this enzyme to cytosine deamination repair**. *J Bacteriol* 1991, **173**(21):6807-6810.
15. Bielawski JP, Gold JR: **Unequal synonymous substitution rates within and between two protein-coding mitochondrial genes**. *Mol Biol Evol* 1996, **13**(6):889-892.

16. Frank AC, Lobry JR: **Asymmetric substitution patterns: a review of possible underlying mutational or selective mechanisms**. *Gene* 1999, **238**(1):65-77.
17. Krishnan NM, Raina SZ, Pollock DD: **Analysis of among-site variation in substitution patterns**. *Biological Procedures Online* 2004, **6**:180-188.
18. Krishnan NM, Seligmann H, Raina SZ, Pollock DD: **Detecting gradients of asymmetry in site-specific substitutions in mitochondrial genomes**. *DNA Cell Biol* 2004, **23**(10):707-714.
19. Rand DM, Kann LM: **Mutation and selection at silent and replacement sites in the evolution of animal mitochondrial DNA**. *Genetica* 1998, **103**:393-407.
