# Supplementary material for: Comparative mitochondrial genomics of snakes: extraordinary substitution rate dynamics and functionality of the duplicate control region
Source: BMC Evol Biol. 2007 Jul 26;7:123. doi: 10.1186/1471-2148-7-123 (PMC1950710; doi:10.1186/1471-2148-7-123)
Supplement: Additional file 2 — Laboratory and genome annotation methods. [file 1471-2148-7-123-S2.pdf]

# Additional File 2 (Supplementary Tables)

**Supplementary Table S1**. Detailed genome annotation of *Agkistrodon piscivorus*.

**Supplementary Table S2**. Detailed genome annotation of *Pantherophis slowinskii*.

**Supplementary Table S3.** Gene-specific polymorphisms observed between the two *Agkistrodon piscivorus* genomes (*Api1* and *Api2*)

**Supplementary Table S4**. Polymorphisms observed in tRNA genes between *Agkistrodon piscivorus* genomes (*Api1* and *Api2*).

**Supplementary Table S5**. Nucleotide frequencies of mitochondrial genome regions (rRNA and protein-coding genes) used in phylogenetic and molecular evolutionary analyses in this study.

**Supplementary Table S6**. Estimated $T_{AMS}$ values of genes for squamates. Two $T_{AMS}$ values are given for each species of alethinophidian snakes; $T_{AMS}^1$ is estimated based on the assumption of exclusive CR1 usage, whereas $T_{AMS}^2$ is estimated based on exclusive CR2 usage. Genes that have alternative $T_{AMS}$ estimates under different CR usage scenarios in alethinophidian mtDNAs are indicated in bold.

**Supplementary Table S7**. Energy (*kcal/mol*) of the cloverleaf structures of tRNAs in snakes and lizards.

**Supplementary Table S8.** Complete mitochondrial genomes used in this study, and associated Genbank accession numbers.

**Supplementary Table S9**. Primer sets used to amplify mitochondrial genome fragments in this study.

**Supplementary Table S1**. Detailed genome annotation of *Agkistrodon piscivorus*.

| | From | To | Size | Strand | Codon | StartCodon | StopCodon |
|---|---|---|---|---|---|---|---|
| **Phe** | 1 | 65 | 65 | L | TTC | | |
| **12sRNA** | 62 | 976 | 915 | - | | | |
| **Val** | 977 | 1040 | 64 | L | GTA | | |
| **16sRNA** | 1041 | 2527 | 1487 | - | | | |
| **ND1** | 2528 | 3488 | 961 | L | | ATC | T |
| **Ile** | 3489 | 3556 | 68 | L | ATC | | |
| **Pro** | 3560 | 3622 | 63 | H | CCA | | |
| **CR1** | 3623 | 4642 | 1020 | - | | | |
| **Leu** | 4643 | 4715 | 73 | L | TTA | | |
| **Gln** | 4716 | 4785 | 70 | H | CAA | | |
| **Met** | 4786 | 4848 | 63 | L | ATG | | |
| **ND2** | 4849 | 5878 | 1030 | L | | ATA | T |
| **Trp** | 5879 | 5944 | 66 | L | TGA | | |
| **Ala** | 5945 | 6009 | 65 | H | GCA | | |
| **Asn** | 6010 | 6081 | 72 | H | AAC | | |
| **$O_L$** | 6084 | 6117 | 34 | - | | | |
| **Cys** | 6116 | 6175 | 60 | H | TGC | | |
| **Tyr** | 6176 | 6236 | 61 | H | TAC | | |
| **COX1** | 6238 | 7839 | 1602 | L | | GTG | AGA |
| **Ser4** | 7830 | 7897 | 68 | H | TCA | | |
| **Asp** | 7898 | 7960 | 63 | L | GAC | | |
| **COX2** | 7962 | 8646 | 685 | L | | ATG | T |
| **Lys** | 8647 | 8710 | 64 | L | AAA | | |
| **ATP8** | 8711 | 8875 | 165 | L | | ATG | TAA |
| **ATP6** | 8866 | 9546 | 681 | L | | ATG | TAA |
| **COX3** | 9546 | 10329 | 784 | L | | ATG | T |
| **Gly** | 10330 | 10390 | 61 | L | GGA | | |
| **ND3** | 10391 | 10733 | 343 | L | | ATC | T |
| **Arg** | 10734 | 10797 | 64 | L | CGA | | |
| **ND4L** | 10798 | 11087 | 290 | L | | ATG | TA |
| **ND4** | 11088 | 12425 | 1338 | L | | ATG | AGA |
| **His** | 12426 | 12487 | 62 | L | CAC | | |
| **Ser2** | 12488 | 12542 | 55 | L | AGC | | |
| **Leu4** | 12543 | 12614 | 72 | L | CTA | | |
| **ND5** | 12616 | 14403 | 1788 | L | | ATG | TAA |
| **ND6** | 14399 | 14908 | 510 | H | | GTG | AGG |
| **Glu** | 14918 | 14980 | 63 | H | GAA | | |
| **CytB** | 14981 | 16094 | 1114 | L | | ATG | T |
| **Thr** | 16095 | 16159 | 65 | L | ACA | | |
| **Pseudo-Pro** | 16160 | 16190 | 31 | - | | | |
| **CR2** | 16191 | 17213 | 1019 | - | | | |

*standard amino acid abbreviations represent the tRNA that codes for that amino acid

**Supplementary Table S2**. Detailed genome annotation of *Pantherophis slowinskii*.

| | From | To | Size (bp) | Strand | Codon | StartCodon | StopCodon |
|---|---|---|---|---|---|---|---|
| **Phe*** | 1 | 60 | 60 | L | TTC | | |
| **12sRNA** | 59 | 991 | 933 | - | | | |
| **Val** | 992 | 1054 | 63 | L | GTA | | |
| **16sRNA** | 1055 | 2531 | 1477 | - | | | |
| **ND1** | 2532 | 3495 | 964 | L | | ATA | T |
| **Ile** | 3496 | 3561 | 66 | L | ATC | | |
| **Pseudo-Pro** | 3558 | 3592 | 35 | | | | |
| **CR1** | 3593 | 4613 | 1021 | - | | | |
| **Leu2** | 4614 | 4686 | 73 | L | TTA | | |
| **Gln** | 4689 | 4759 | 71 | H | CAA | | |
| **Met** | 4761 | 4822 | 62 | L | ATG | | |
| **ND2** | 4823 | 5852 | 1030 | L | | ATT | T |
| **Trp** | 5853 | 5917 | 65 | L | TGA | | |
| **Ala** | 5919 | 5981 | 63 | H | GCA | | |
| **Asn** | 5983 | 6055 | 73 | H | AAC | | |
| **$O_L$** | 6058 | 6093 | 36 | - | | | |
| **Cys** | 6092 | 6152 | 61 | H | TGC | | |
| **Tyr** | 6153 | 6214 | 62 | H | TAC | | |
| **COX1** | 6216 | 7817 | 1602 | L | | GTG | AGA |
| **Ser4** | 7808 | 7874 | 67 | H | TCA | | |
| **Asp** | 7875 | 7938 | 64 | L | GAC | | |
| **COX2** | 7940 | 8624 | 685 | L | | ATG | T |
| **Lys** | 8625 | 8688 | 64 | L | AAA | | |
| **ATP8** | 8690 | 8848 | 159 | L | | ATG | TAA |
| **ATP6** | 8839 | 9519 | 681 | L | | ATG | TAA |
| **COX3** | 9519 | 10302 | 784 | L | | ATG | T |
| **Gly** | 10303 | 10363 | 61 | L | GGA | | |
| **ND3** | 10364 | 10706 | 343 | L | | GTG | T |
| **Arg** | 10707 | 10771 | 65 | L | CGA | | |
| **ND4L** | 10772 | 11061 | 290 | L | | ATG | TA |
| **ND4** | 11062 | 12399 | 1338 | L | | ATG | TAA |
| **His** | 12400 | 12464 | 65 | L | CAC | | |
| **Ser2** | 12465 | 12521 | 57 | L | AGC | | |
| **Leu4** | 12519 | 12589 | 71 | L | CTA | | |
| **ND5** | 12590 | 14536 | 1947 | L | | ATG | ATT |
| **ND6** | 14353 | 14853 | 501 | H | | ATG | TAG |
| **Glu** | 14863 | 14924 | 62 | H | GAA | | |
| **CytB** | 14923 | 16039 | 1117 | L | | ATG | T |
| **Thr** | 16040 | 16103 | 64 | L | ACA | | |
| **Pro** | 16104 | 16164 | 61 | H | CCA | | |
| **CR2** | 16165 | 17189 | 1025 | - | | | |

*standard amino acid abbreviations represent the tRNA that codes for that amino acid

**Supplementary Table S3.** Gene-specific polymorphisms observed between the two *Agkistrodon piscivorus* genomes (*Api1* and *Api2*)

| | | | Substitutions | | | | |
|---|---|---|---|---|---|---|---|
| **Genes** | **Length** | **Similarity** | **all** | **1st** | **2nd** | **3rd** | **AA** |
| **12s RNA** | 915 | 98.80% | 11 | - | - | - | - |
| **16s RNA** | 1487 | 97.40% | 39 | - | - | - | - |
| **ATP6** | 681 | 95.00% | 32 | 5 | 2 | 25 | 4 |
| **ATP8** | 165 | 93.94% | 11 | 3 | 1 | 7 | 3 |
| **COX1** | 1602 | 96.38% | 58 | 0 | 1 | 57 | 2 |
| **COX2** | 685 | 96.50% | 24 | 6 | 0 | 18 | 3 |
| **COX3** | 786 | 96.40% | 28 | 6 | 1 | 21 | 5 |
| **CytB** | 1114 | 95.33% | 52 | 10 | 3 | 39 | 10 |
| **ND1** | 960 | 96.46% | 34 | 8 | 1 | 25 | 3 |
| **ND2** | 1030 | 96.12% | 40 | 6 | 4 | 30 | 8 |
| **ND3** | 343 | 93.88% | 21 | 2 | 6 | 20 | 8 |
| **ND4** | 1338 | 95.81% | 56 | 9 | 3 | 44 | 5 |
| **ND4L** | 290 | 97.93% | 6 | 2 | 0 | 4 | 2 |
| **ND5** | 1788 | 94.46% | 96 | 21 | 9 | 69 | 28 |
| **ND6** | 510 | 95.00% | 26 | 3 | 4 | 19 | 5 |
| **CR1** | 1021 | 98.20% | 19 | - | - | - | - |
| **CR2** | 1022 | 98.40% | 18 | - | - | - | - |

**Supplementary Table S4**. Polymorphisms observed in tRNA genes between *Agkistrodon piscivorus* genomes (*Api1* and *Api2*).

| tRNA | Length | Similarity | Substitution location |
|---|---|---|---|
| ***Phe*** | 65 | 96.92% | deletion of g in D-Loop and t-c in T-loop |
| ***Val*** | 64 | 98% | t-c in T-Loop |
| ***Ile*** | 68 | 92.65% | a-g g-a,c-t,t-c in T-Loop, and a-g in stem |
| ***Pro*** | 63 | 100% | |
| ***Leu*** | 73 | 100% | |
| ***Gln*** | 70 | 100% | |
| ***Met*** | 63 | 100% | deletion of a in D-arm |
| ***Trp*** | 66 | 95.45% | g-a and a-g in anticodon arm, and g-t in T-Loop |
| ***Ala*** | 65 | 98.46% | c-t in variable loop |
| ***Asn*** | 72 | 100% | |
| ***Cys*** | 60 | 96.67% | c-t in stem, t-c in T-Loop |
| ***Tyr*** | 61 | 100% | |
| ***Ser4*** | 68 | 98.53% | t-g in D-Loop |
| ***Asp*** | 63 | 100% | |
| ***Lys*** | 64 | 98.44% | deletion of t in T-Loop |
| ***Gly*** | 61 | 100% | deletion of a in D-arm |
| ***Arg*** | 64 | 98.44% | a-g in stem |
| ***His*** | 62 | 98.39% | c-t in stem |
| ***Ser2*** | 55 | 98.18% | t-g in D-Loop |
| ***Leu4*** | 72 | 94.44% | c-t in stem, insertion of c in variable loop, a-g in anticodon stem, a-t in T-Loop |
| ***Glu*** | 63 | 93.65% | t-g in D-stem, a-t, t-a and deletion of g in T-Loop |
| ***Thr*** | 65 | 100% | |

**Supplementary Table S5**. Nucleotide frequencies of mitochondrial genome regions (rRNA and protein-coding genes) used in phylogenetic and molecular evolutionary analyses in this study.

| | | Entire Alignment | | | | rRNA Genes | | | | Protein-Coding Genes | | | |
|---|---|---|---|---|---|---|---|---|---|---|---|---|---|
| | Species | A | C | G | T | A | C | G | T | A | C | G | T |
| Amphibians | *Mertensiella luschani* | 0.31 | 0.24 | 0.15 | 0.3 | 0.39 | 0.19 | 0.17 | 0.25 | 0.3 | 0.25 | 0.15 | 0.31 |
| | *Xenopus laevis* | 0.31 | 0.24 | 0.14 | 0.31 | 0.35 | 0.23 | 0.18 | 0.24 | 0.31 | 0.24 | 0.13 | 0.32 |
| Mammals | *Bos taurus* | 0.33 | 0.26 | 0.14 | 0.28 | 0.37 | 0.22 | 0.18 | 0.23 | 0.31 | 0.27 | 0.13 | 0.29 |
| | *Cebus albifrons* | 0.32 | 0.26 | 0.13 | 0.29 | 0.35 | 0.24 | 0.17 | 0.24 | 0.31 | 0.27 | 0.12 | 0.3 |
| | *Gorilla gorilla* | 0.3 | 0.3 | 0.14 | 0.26 | 0.35 | 0.26 | 0.18 | 0.22 | 0.29 | 0.31 | 0.13 | 0.27 |
| | *Homo sapiens* | 0.3 | 0.31 | 0.14 | 0.25 | 0.34 | 0.26 | 0.18 | 0.22 | 0.29 | 0.32 | 0.13 | 0.26 |
| | *Hylobates lar* | 0.3 | 0.31 | 0.14 | 0.25 | 0.34 | 0.27 | 0.18 | 0.21 | 0.29 | 0.32 | 0.14 | 0.25 |
| | *Lemur catta* | 0.32 | 0.25 | 0.13 | 0.3 | 0.36 | 0.23 | 0.17 | 0.24 | 0.31 | 0.25 | 0.12 | 0.31 |
| | *Macaca sylvanus* | 0.31 | 0.3 | 0.14 | 0.26 | 0.35 | 0.25 | 0.18 | 0.22 | 0.3 | 0.31 | 0.13 | 0.27 |
| | *Nycticebus coucang* | 0.31 | 0.27 | 0.15 | 0.28 | 0.36 | 0.23 | 0.18 | 0.23 | 0.3 | 0.27 | 0.14 | 0.29 |
| | *Pan paniscus* | 0.3 | 0.3 | 0.13 | 0.26 | 0.35 | 0.26 | 0.18 | 0.22 | 0.29 | 0.31 | 0.13 | 0.27 |
| | *Papio hamadryas* | 0.3 | 0.3 | 0.14 | 0.26 | 0.34 | 0.25 | 0.18 | 0.23 | 0.3 | 0.31 | 0.13 | 0.26 |
| | *Pongo pygmaeus* | 0.3 | 0.32 | 0.14 | 0.24 | 0.34 | 0.26 | 0.19 | 0.21 | 0.29 | 0.33 | 0.13 | 0.25 |
| | *Tarsius bancanus* | 0.32 | 0.26 | 0.13 | 0.29 | 0.37 | 0.23 | 0.17 | 0.24 | 0.31 | 0.27 | 0.12 | 0.3 |
| Tuatara | *Sphenodon punctatus* | 0.32 | 0.26 | 0.15 | 0.27 | 0.37 | 0.23 | 0.18 | 0.22 | 0.31 | 0.26 | 0.14 | 0.29 |
| Lizards | *Abronia graminea* | 0.33 | 0.27 | 0.14 | 0.27 | 0.38 | 0.24 | 0.17 | 0.21 | 0.31 | 0.27 | 0.13 | 0.29 |
| | *Cordylus warreni* | 0.3 | 0.3 | 0.14 | 0.25 | 0.35 | 0.26 | 0.18 | 0.21 | 0.29 | 0.31 | 0.14 | 0.27 |
| | *Eumeces egregius* | 0.29 | 0.28 | 0.16 | 0.26 | 0.35 | 0.25 | 0.19 | 0.21 | 0.28 | 0.29 | 0.16 | 0.27 |
| | *Iguana iguana* | 0.3 | 0.32 | 0.14 | 0.23 | 0.35 | 0.28 | 0.18 | 0.19 | 0.29 | 0.33 | 0.14 | 0.24 |
| | *Sceloporus occidentalis* | 0.32 | 0.28 | 0.15 | 0.25 | 0.37 | 0.25 | 0.18 | 0.21 | 0.31 | 0.29 | 0.14 | 0.26 |
| | *Shinisaurus crocodilurus* | 0.31 | 0.27 | 0.14 | 0.28 | 0.37 | 0.24 | 0.17 | 0.23 | 0.29 | 0.28 | 0.14 | 0.29 |
| | *Varanus komodoensis* | 0.29 | 0.31 | 0.14 | 0.27 | 0.34 | 0.29 | 0.17 | 0.2 | 0.28 | 0.31 | 0.13 | 0.28 |
| Snakes | *Acrochordus granulatus* | 0.35 | 0.25 | 0.12 | 0.28 | 0.39 | 0.24 | 0.15 | 0.21 | 0.34 | 0.25 | 0.11 | 0.3 |
| | *Agkistrodon piscivorus (Api1)* | 0.32 | 0.29 | 0.14 | 0.25 | 0.37 | 0.25 | 0.17 | 0.21 | 0.31 | 0.3 | 0.13 | 0.26 |
| | *Agkistrodon piscivorus (Api2)* | 0.32 | 0.29 | 0.14 | 0.25 | 0.37 | 0.24 | 0.17 | 0.21 | 0.31 | 0.31 | 0.13 | 0.25 |
| | *Boa constrictor* | 0.36 | 0.26 | 0.14 | 0.24 | 0.41 | 0.23 | 0.17 | 0.19 | 0.35 | 0.27 | 0.13 | 0.25 |
| | *Cylindrophis ruffus* | 0.35 | 0.27 | 0.13 | 0.25 | 0.39 | 0.25 | 0.16 | 0.2 | 0.34 | 0.27 | 0.12 | 0.27 |
| | *Dinodon semicarinatus* | 0.35 | 0.27 | 0.13 | 0.26 | 0.39 | 0.25 | 0.16 | 0.2 | 0.34 | 0.27 | 0.12 | 0.27 |
| | *Leptotyphlops dulcis* | 0.34 | 0.31 | 0.13 | 0.21 | 0.36 | 0.29 | 0.18 | 0.17 | 0.33 | 0.32 | 0.12 | 0.22 |
| | *Ovophis okinavensis* | 0.32 | 0.29 | 0.13 | 0.26 | 0.38 | 0.24 | 0.16 | 0.22 | 0.31 | 0.3 | 0.13 | 0.26 |
| | *Pantherophis slowinskii* | 0.35 | 0.25 | 0.13 | 0.26 | 0.39 | 0.23 | 0.16 | 0.21 | 0.34 | 0.26 | 0.13 | 0.28 |
| | *Python regius* | 0.33 | 0.29 | 0.13 | 0.25 | 0.38 | 0.26 | 0.16 | 0.2 | 0.32 | 0.3 | 0.13 | 0.26 |
| | *Xenopeltis unicolor* | 0.34 | 0.27 | 0.13 | 0.26 | 0.38 | 0.24 | 0.16 | 0.21 | 0.33 | 0.27 | 0.13 | 0.27 |
| Turtles | *Chelonia mydas* | 0.34 | 0.28 | 0.12 | 0.26 | 0.38 | 0.23 | 0.17 | 0.22 | 0.33 | 0.29 | 0.12 | 0.27 |
| | *Chrysemys picta* | 0.33 | 0.26 | 0.13 | 0.27 | 0.39 | 0.23 | 0.17 | 0.21 | 0.32 | 0.27 | 0.13 | 0.28 |
| | *Dogania subplana* | 0.34 | 0.26 | 0.13 | 0.26 | 0.39 | 0.23 | 0.16 | 0.22 | 0.33 | 0.27 | 0.12 | 0.27 |
| | *Pelomedusa subrufa* | 0.32 | 0.27 | 0.13 | 0.28 | 0.36 | 0.24 | 0.17 | 0.23 | 0.32 | 0.28 | 0.12 | 0.29 |
| Crocodilians | *Alligator mississsippiensis* | 0.3 | 0.29 | 0.14 | 0.26 | 0.35 | 0.26 | 0.18 | 0.21 | 0.29 | 0.3 | 0.13 | 0.27 |
| | *Alligator sinensis* | 0.28 | 0.31 | 0.16 | 0.25 | 0.33 | 0.27 | 0.2 | 0.2 | 0.27 | 0.32 | 0.15 | 0.26 |
| | *Caiman crocodilus* | 0.31 | 0.31 | 0.14 | 0.25 | 0.34 | 0.27 | 0.19 | 0.2 | 0.3 | 0.32 | 0.13 | 0.26 |
| Birds | *Apteryx haastii* | 0.3 | 0.3 | 0.14 | 0.26 | 0.35 | 0.25 | 0.18 | 0.22 | 0.29 | 0.31 | 0.13 | 0.27 |
| | *Buteo buteo* | 0.29 | 0.33 | 0.14 | 0.24 | 0.33 | 0.28 | 0.19 | 0.21 | 0.28 | 0.34 | 0.13 | 0.24 |
| | *Ciconia boyciana* | 0.29 | 0.33 | 0.15 | 0.23 | 0.33 | 0.28 | 0.19 | 0.2 | 0.28 | 0.34 | 0.14 | 0.24 |
| | *Ciconia ciconia* | 0.29 | 0.33 | 0.15 | 0.23 | 0.33 | 0.28 | 0.19 | 0.2 | 0.28 | 0.34 | 0.14 | 0.24 |
| | *Corvus frugilegus* | 0.3 | 0.3 | 0.15 | 0.25 | 0.33 | 0.24 | 0.21 | 0.22 | 0.29 | 0.31 | 0.14 | 0.26 |
| | *Dromaius novaehollandiae* | 0.3 | 0.29 | 0.14 | 0.27 | 0.35 | 0.24 | 0.19 | 0.22 | 0.29 | 0.3 | 0.13 | 0.28 |
| | *Falco peregrinus* | 0.3 | 0.32 | 0.14 | 0.24 | 0.34 | 0.28 | 0.19 | 0.19 | 0.29 | 0.33 | 0.13 | 0.25 |
| | *Gallus gallus* | 0.29 | 0.33 | 0.14 | 0.24 | 0.33 | 0.28 | 0.18 | 0.2 | 0.28 | 0.34 | 0.13 | 0.25 |
| | *Rhea americana* | 0.27 | 0.33 | 0.15 | 0.25 | 0.33 | 0.27 | 0.19 | 0.21 | 0.26 | 0.34 | 0.15 | 0.26 |
| | *Smithornis sharpei* | 0.29 | 0.33 | 0.14 | 0.25 | 0.33 | 0.29 | 0.18 | 0.2 | 0.28 | 0.33 | 0.13 | 0.26 |
| | *Struthio camelus* | 0.29 | 0.3 | 0.15 | 0.26 | 0.34 | 0.26 | 0.18 | 0.21 | 0.28 | 0.31 | 0.14 | 0.27 |
| | *Tinamus major* | 0.29 | 0.3 | 0.14 | 0.27 | 0.34 | 0.26 | 0.18 | 0.22 | 0.28 | 0.31 | 0.13 | 0.28 |
| | *Vidua chalybeata* | 0.3 | 0.31 | 0.16 | 0.23 | 0.33 | 0.25 | 0.21 | 0.21 | 0.3 | 0.32 | 0.15 | 0.24 |
| | **Mean** | **0.31** | **0.29** | **0.14** | **0.26** | **0.36** | **0.25** | **0.18** | **0.21** | **0.3** | **0.3** | **0.13** | **0.27** |

**Supplementary Table S6**. Estimated $T_{AMS}$ values of genes for squamates. Two $T_{AMS}$ values are given for each species of alethinophidian snakes; $T_{AMS}^{1}$ is estimated based on the assumption of exclusive CR1 usage, whereas $T_{AMS}^{2}$ is estimated based on exclusive CR2 usage. Genes that have alternative $T_{AMS}$ estimates under different CR usage scenarios in alethinophidian mtDNAs are indicated in bold.

| | Snakes | | | | | | | | | | | | | | | | | | | Lizards | | | | | |
|---|---|---|---|---|---|---|---|---|---|---|---|---|---|---|---|---|---|---|---|---|---|---|---|---|---|
| | *Agkistrodon* | | *Ovophis* | | *Pantherophis* | | *Dinodon* | | *Acrochordus* | | *Boa* | | *Cylindrophis* | | *Python* | | *Xenopeltis* | | *Leptotyphlops* | *Iguana* | *Eumeces* | *Sceloporus* | *Cordylus* | *Abronia* | *Shinisaurus* |
| **Genes** | $T_{AMS}1$ | $T_{AMS}2$ | $T_{AMS}1$ | $T_{AMS}2$ | $T_{AMS}1$ | $T_{AMS}2$ | $T_{AMS}1$ | $T_{AMS}2$ | $T_{AMS}1$ | $T_{AMS}2$ | $T_{AMS}1$ | $T_{AMS}2$ | $T_{AMS}1$ | $T_{AMS}2$ | $T_{AMS}1$ | $T_{AMS}2$ | $T_{AMS}1$ | $T_{AMS}2$ | $T_{AMS}$ | $T_{AMS}$ | $T_{AMS}$ | $T_{AMS}$ | $T_{AMS}$ | $T_{AMS}$ | $T_{AMS}$ |
| **12s** | **0.35** | **1.36** | **0.34** | **1.34** | **0.35** | **1.35** | **0.35** | **1.35** | **0.35** | **1.35** | **0.33** | **1.33** | **0.35** | **1.35** | **0.36** | **1.36** | **0.32** | **1.32** | 0.45 | 0.44 | 0.47 | 0.46 | 0.47 | 0.43 | 0.45 |
| **16s** | **0.50** | **1.51** | **0.48** | **1.48** | **0.50** | **1.50** | **0.50** | **1.49** | **0.50** | **1.49** | **0.47** | **1.46** | **0.50** | **1.49** | **0.51** | **1.50** | **0.46** | **1.45** | 0.61 | 0.60 | 0.62 | 0.62 | 0.62 | 0.59 | 0.60 |
| **ATP6** | 0.36 | 0.36 | 0.36 | 0.36 | 0.36 | 0.36 | 0.36 | 0.36 | 0.35 | 0.35 | 0.33 | 0.33 | 0.35 | 0.35 | 0.36 | 0.36 | 0.33 | 0.33 | 0.39 | 0.37 | 0.35 | 0.36 | 0.36 | 0.39 | 0.37 |
| **ATP8** | 0.31 | 0.31 | 0.31 | 0.31 | 0.31 | 0.31 | 0.31 | 0.31 | 0.31 | 0.31 | 0.29 | 0.29 | 0.31 | 0.31 | 0.31 | 0.31 | 0.29 | 0.29 | 0.34 | 0.32 | 0.31 | 0.31 | 0.31 | 0.33 | 0.32 |
| **COX1** | 0.11 | 0.11 | 0.11 | 0.11 | 0.11 | 0.11 | 0.11 | 0.11 | 0.11 | 0.11 | 0.10 | 0.10 | 0.10 | 0.10 | 0.11 | 0.11 | 0.10 | 0.10 | 0.12 | 0.11 | 0.11 | 0.11 | 0.11 | 0.12 | 0.11 |
| **COX2** | 0.26 | 0.26 | 0.25 | 0.25 | 0.26 | 0.26 | 0.26 | 0.26 | 0.25 | 0.25 | 0.23 | 0.23 | 0.25 | 0.25 | 0.26 | 0.26 | 0.23 | 0.23 | 0.28 | 0.26 | 0.25 | 0.26 | 0.25 | 0.27 | 0.26 |
| **COX3** | 0.45 | 0.45 | 0.44 | 0.44 | 0.45 | 0.45 | 0.45 | 0.45 | 0.44 | 0.44 | 0.41 | 0.41 | 0.44 | 0.44 | 0.45 | 0.45 | 0.41 | 0.41 | 0.48 | 0.46 | 0.44 | 0.45 | 0.45 | 0.48 | 0.46 |
| **CytB** | 1.10 | 1.10 | 1.08 | 1.08 | 1.09 | 1.09 | 1.09 | 1.09 | 1.07 | 1.07 | 1.00 | 1.00 | 1.08 | 1.08 | 1.10 | 1.10 | 1.01 | 1.01 | 1.17 | 1.15 | 1.10 | 1.12 | 1.11 | 1.19 | 1.15 |
| **ND1** | **0.64** | **1.65** | **0.62** | **1.62** | **0.64** | **1.64** | **0.64** | **1.63** | **0.64** | **1.64** | **0.60** | **1.60** | **0.64** | **1.64** | **0.66** | **1.66** | **0.59** | **1.59** | 0.77 | 0.76 | 0.78 | 0.77 | 0.77 | 0.76 | 0.76 |
| **ND2** | 0.91 | 0.91 | 0.92 | 0.92 | 0.91 | 0.91 | 0.91 | 0.91 | 0.92 | 0.92 | 0.92 | 0.92 | 0.92 | 0.92 | 0.91 | 0.91 | 0.92 | 0.92 | 0.91 | 0.91 | 0.91 | 0.91 | 0.91 | 0.91 | 0.91 |
| **ND3** | 0.52 | 0.52 | 0.51 | 0.51 | 0.52 | 0.52 | 0.52 | 0.52 | 0.51 | 0.51 | 0.47 | 0.47 | 0.51 | 0.51 | 0.52 | 0.52 | 0.47 | 0.47 | 0.55 | 0.54 | 0.51 | 0.52 | 0.52 | 0.56 | 0.54 |
| **ND4** | 0.66 | 0.66 | 0.65 | 0.65 | 0.66 | 0.66 | 0.66 | 0.66 | 0.64 | 0.64 | 0.60 | 0.60 | 0.65 | 0.65 | 0.66 | 0.66 | 0.60 | 0.60 | 0.70 | 0.68 | 0.65 | 0.67 | 0.66 | 0.71 | 0.68 |
| **ND4L** | 0.56 | 0.56 | 0.56 | 0.56 | 0.56 | 0.56 | 0.56 | 0.56 | 0.55 | 0.55 | 0.51 | 0.51 | 0.55 | 0.55 | 0.56 | 0.56 | 0.52 | 0.52 | 0.60 | 0.58 | 0.56 | 0.57 | 0.56 | 0.60 | 0.58 |
| **ND5** | 0.86 | 0.86 | 0.85 | 0.85 | 0.86 | 0.86 | 0.86 | 0.86 | 0.84 | 0.84 | 0.79 | 0.79 | 0.85 | 0.85 | 0.86 | 0.86 | 0.79 | 0.79 | 0.92 | 0.90 | 0.86 | 0.88 | 0.87 | 0.93 | 0.90 |
| **ND6** | 0.99 | 0.99 | 0.98 | 0.98 | 0.99 | 0.99 | 0.99 | 0.99 | 0.97 | 0.97 | 0.91 | 0.91 | 0.98 | 0.98 | 1.00 | 1.00 | 0.91 | 0.91 | 1.06 | 1.04 | 0.99 | 1.01 | 1.01 | 1.08 | 1.04 |

**Supplementary Table S7**. Energy (*kcal/mol*) of the cloverleaf structures of tRNAs in snakes and lizards.

| | Snakes | | | | | | | | | | Lizards and Tuatara | | | | | | | |
|---|---|---|---|---|---|---|---|---|---|---|---|---|---|---|---|---|---|---|
| | ***Agkistrodon piscivorus*** | ***Pantherophis guttatus*** | ***Dinodon semicarinatus*** | ***Acrochordus granulatus*** | ***Boa constrictor*** | ***Ovophis okinavensis*** | ***Cylindrophis ruffus*** | ***Python regius*** | ***Xenopeltis unicolor*** | ***Leptotyphlops dulcis*** | ***Iguana iguana*** | ***Eumeces egregius*** | ***Sceloporus occidentalis*** | ***Cordylus warreni*** | ***Abronia graminea*** | ***Shinisaurus crocodilurus*** | ***Varanus komodoensis*** | ***Sphenodon punctatus*** |
| **Ala** | -10 | -4.8 | -8.9 | -6.6 | -13 | -10 | -11 | -9.5 | -8.5 | -15 | -12 | -7.3 | -11 | -8.5 | -8.1 | -8.2 | -8.2 | -7.6 |
| **Arg** | -7.3 | -3.5 | -7 | -14 | -11 | -9.6 | -10 | -11 | -14 | -5.4 | -18 | -6.5 | -17 | -12 | -15 | -12 | -19 | -11 |
| **Asn** | -14 | -21 | -12 | -13 | -20 | -14 | -16 | -13 | -20 | -17 | -13 | -15 | -20 | -18 | -14 | -15 | -19 | -15 |
| **Asp** | -16 | -13 | -14 | -9.3 | -27 | -19 | -20 | -13 | -11 | -8.9 | -9 | -16 | -15 | -16 | -15 | -13 | -6.9 | -7.2 |
| **Cys** | -19 | -18 | -18 | -18 | -24 | -19 | -15 | -24 | -24 | -14 | -19 | -19 | -20 | -13 | -16 | -17 | -19 | -3.8 |
| **Gln** | -10 | -10 | -8.9 | -7.4 | -13 | -10 | -12 | -15 | -13 | -13 | -14 | -16 | -14 | -8.6 | -13 | -13 | -2.5 | -14 |
| **Glu** | -12 | -11 | -8.7 | -9 | -11 | -13 | -13 | -15 | -11 | -11 | -12 | -11 | -9.4 | -11 | -16 | -13 | -12 | -14 |
| **Gly** | -6.9 | -4.9 | -10 | -7.4 | -9.2 | -7 | -11 | -12 | -11 | -7.2 | -11 | -15 | -9 | -14 | -12 | -12 | -17 | -9.3 |
| **His** | -5.8 | -5.2 | -9.4 | -4.2 | -7.6 | -3.5 | -6.2 | -8.6 | -7.6 | -8.8 | -9.3 | -11 | -8.6 | -9.6 | -1.2 | -7.9 | -9.4 | N/A |
| **Ile** | -15 | -15 | -17 | -14 | -15 | -11 | -16 | -15 | -15 | -8.6 | -7.9 | -14 | -13 | -17 | -13 | -10 | -15 | -13 |
| **Leu2** | -10 | -8.7 | -10 | 6.1 | -9 | -11 | -8.3 | -12 | -9 | -18 | -18 | -10 | -14 | -13 | -12 | -13 | -9.3 | -12 |
| **Leu4** | -20 | -15 | -16 | -16 | -17 | -16 | -14 | -15 | -16 | -13 | -15 | -17 | -14 | -12 | -16 | -13 | -15 | -25 |
| **Lys** | -11 | -15 | -13 | -12 | -14 | -13 | -11 | -11 | -10 | -19 | -19 | -17 | -19 | -16 | -16 | -15 | -14 | -16 |
| **Met** | -14 | -16 | -16 | 14.4 | -14 | -11 | -13 | -14 | -14 | -13 | -9.3 | -9 | -9.1 | N/a | -9 | -9.4 | -16 | -9.9 |
| **Phe** | -3.8 | -9.6 | -0.3 | -8.7 | -12 | -11 | -7.5 | -12 | -7.6 | -15 | -17 | -17 | -12 | -8.1 | -11 | -15 | -11 | -9.9 |
| **Pro** | -13 | -8.5 | -8.7 | -6.5 | -8.2 | -14 | -6.6 | -9.8 | -5.8 | -11 | -15 | -13 | -15 | -11 | -14 | -6.6 | -9.6 | -19 |
| **Ser4** | -14 | -13 | -15 | -13 | -17 | -10 | -16 | -11 | -18 | -19 | -14 | -23 | -14 | -16 | -16 | -14 | -14 | -8.6 |
| **Thr** | -7.5 | -10 | -4.6 | -11 | -8.2 | -8.7 | -10 | -11 | -6.5 | -12 | -14 | -8 | -14 | -13 | -12 | -18 | -15 | N/A |
| **Trp** | -8.3 | -7.7 | -6.6 | -5.2 | -5.3 | -8.5 | -5.4 | -7.4 | -4.9 | -7.4 | -8.6 | -21 | -22 | -13 | -17 | -5.5 | -5.9 | -16 |
| **Tyr** | -15 | -11 | -12 | -16 | -16 | -15 | -14 | -18 | -11 | -15 | -16 | -29 | -16 | -21 | -16 | -14 | -14 | -14 |
| **Val** | -7.4 | -8.8 | -6.7 | -7.1 | -7.6 | -7.5 | -7.2 | -7.5 | -8.3 | -3.7 | -9.2 | -6.4 | -6.9 | -7.1 | -5.6 | -6.9 | -10 | -11 |

**Supplementary Table S8.** Complete mitochondrial genomes used in this study, and associated Genbank accession numbers.

| Vertebrate Group | Genbank Accession | Taxon | Vertebrate Group | Genbank Accession | Taxon |
|---|---|---|---|---|---|
| Amphibians | NC_002756 | *Mertensiella luschani* | Birds | NC_002782 | *Apteryx haastii* |
| | NC_001573 | *Xenopus laevis* | | NC_003128 | *Buteo buteo* |
| Turtles | NC_000886 | *Chelonia mydas* | | NC_002196 | *Ciconia boyciana* |
| | NC_002073 | *Chrysemys picta* | | NC_002197 | *Ciconia ciconia* |
| | NC_002780 | *Dogania subplana* | | NC_002069 | *Corvus frugilegus* |
| | NC_001947 | *Pelomedusa subrufa* | | NC_002784 | *Dromaius novaehollandiae* |
| Tuatara | NC_004815 | *Sphenodon punctatus* | | NC_000878 | *Falco peregrinus* |
| Lizards | NC_005958 | *Abronia graminea* | | NC_001323 | *Gallus gallus* |
| | NC_005962 | *Cordylus warreni* | | NC_000846 | *Rhea americana* |
| | NC_000888 | *Eumeces egregius* | | NC_000879 | *Smithornis sharpei* |
| | NC_002793 | *Iguana iguana* | | NC_002785 | *Struthio camelus* |
| | NC_005960 | *Sceloporus occidentalis* | | NC_002781 | *Tinamus major* |
| | NC_005959 | *Shinisaurus crocodilurus* | | NC_000880 | *Vidua chalybeata* |
| | AB080275-6 | *Varanus komodoensis* | Mammals | NC_001567 | *Bos taurus* |
| Snakes | NC_007400 | *Acrochordus granulatus* | | NC_002763 | *Cebus albifrons* |
| | DQ523162 | *Agkistrodon piscivorus (Api1)* | | NC_002082 | *Hylobates lar* |
| | | | | NC_001646 | *Pongo pygmaeus* |
| | EF669477 | *Agkistrodon piscivorus (Api2)* | | NC_001644 | *Pan paniscus* |
| | | | | NC_001645 | *Gorilla gorilla* |
| | NC_007398 | *Boa constrictor* | | NC_001807 | *Homo sapiens* |
| | NC_007401 | *Cylindrophis ruffus* | | NC_001992 | *Papio hamadryas* |
| | NC_001945 | *Dinodon semicarinatus* | | NC_002764 | *Macaca sylvanus* |
| | NC_005961 | *Leptotyphlops dulcis* | | NC_002811 | *Tarsius bancanus* |
| | NC_007397 | *Ovophis okinavensis* | | NC_004025 | *Lemur catta* |
| | DQ523161 | *Pantherophis slowinskii* | | NC_002765 | *Nycticebus coucang* |
| | NC_007399 | *Python regius* | | | |
| | NC_007402 | *Xenopeltis unicolor* | | | |

**Supplementary Table S9**. Primer sets used to amplify mitochondrial genome fragments in this study.

| Primer Name | Primer sequence (5' – 3') | Source |
|---|---|---|
| ***Agkistrodon piscivorus - Api2* amplification primers** | | |
| L2932 | MYTGGTGCCAGCCGCCGCGG | This study |
| tRNATrpR | GGCTTTGAAGGCTMCTAGTTT | R. Lawson, unpub. |
| | | |
| ND1L | CTATCCCCCATCATAGCMC | This study |
| ND2H | TCGGGGTATGGGCCCG | This study |
| | | |
| LRattle | ACTCTAACGCTCCTAACCTGAC | K. Zamudio, unpub. |
| Leu | CCAACACCTVTTCTGATT | Arévalo et al. 1994 |
| | | |
| L6929 | CCAACACCTVTTCTGATT | This study |
| ND4CP200 | ARATTGYRGCTRCTACTARGCC | This study |
| | | |
| ND4 | CACCTATGACTACCAAAAGCTCATGTAGAAGC | Arévalo et al. 1994 |
| AtrCB3 | TGAGAAGTTTTCYGGGTCRTT | Parkinson et al. 2002 |
| | | |
| Gludg | TGACTTGAARAACCAYCGTTG | Parkinson et al. 2002 |
| H3059 | CCGGTCTGAACTCAGATCACGT | This study |
| ***Agkistrodon piscivorus - Api1* amplification primers** | | |
| DPFB002R | AGTGGTCAWGGGCTKGGGACTA | This study |
| DPFB0013F | CGGCCGCGGTATYCTAACCGTGCAAAG | This study |
| | | |
| DPFB001F | TAGTAGACCCMAGCCCWTGACCACT | This study |
| DPFB0021R | CTGATCCAACATCGAGGTCGTAAACC | This study |
| ***Pantherophis slowinskii* amplification primers** | | |
| DPAL007 | CTACGTGATCTGAGTTCAGACC | This study |
| DPFB007 | CTCAGAAKGATATYTGTCCYCATGG | This study |
| | | |
| DPFB006 | CCATGRGGACARATATCMTTCTGAG | This study |
| DPAL006 | CTCCGGTCTGAACTCAGATCAC | This study |
