# Supplementary material for: Comparative mitochondrial genomics of snakes: extraordinary substitution rate dynamics and functionality of the duplicate control region
Source: BMC Evol Biol. 2007 Jul 26;7:123. doi: 10.1186/1471-2148-7-123 (PMC1950710; doi:10.1186/1471-2148-7-123)
Supplement: Additional file 3 — Comparison of gene lengths in snakes and other squamates. [file 1471-2148-7-123-S3.pdf]

Additional File 3 (Supplementary Figure)

Supplementary Figure S1 - Comparison of mitochondrial gene lengths among snakes and other tetrapod groups.

The total length (b.p.) is shown for all protein coding regions (A), tRNAs (B), and rRNAs (C). All snakes are in gray, while other squamates (lizards) are in black; light gray and dark gray bars are drawn under snakes species to indicate membership in the Colubroidea or Acro-Heno Clade, respectively.

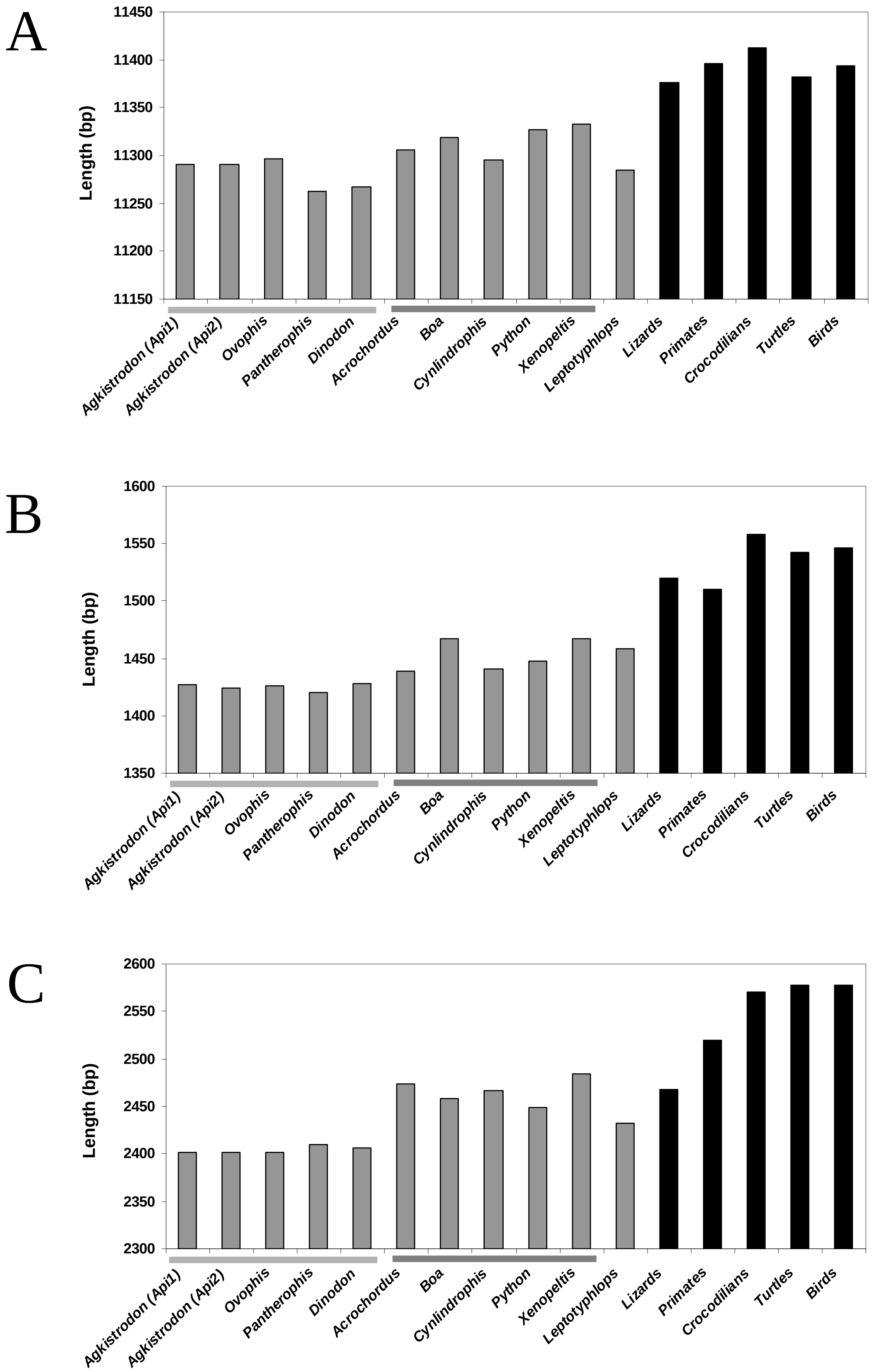
A
Length (bp)
11450
11400
11350
11300
11250
11200
11150
Agkistrodon (Api1)
Agkistrodon (Api2)
Ovophis
Pantherophis
Dinodon
Acrochordus
Boa
Cynlindrophis
Python
Xenopeltis
Leptotyphlops
Lizards
Primates
Crocodilians
Turtles
Birds
B
Length (bp)
1600
1550
1500
1450
1400
1350
Agkistrodon (Api1)
Agkistrodon (Api2)
Ovophis
Pantherophis
Dinodon
Acrochordus
Boa
Cynlindrophis
Python
Xenopeltis
Leptotyphlops
Lizards
Primates
Crocodilians
Turtles
Birds
C
Length (bp)
2600
2550
2500
2450
2400
2350
2300
Agkistrodon (Api1)
Agkistrodon (Api2)
Ovophis
Pantherophis
Dinodon
Acrochordus
Boa
Cynlindrophis
Python
Xenopeltis
Leptotyphlops
Lizards
Primates
Crocodilians
Turtles
Birds
