# Supplementary material for: Comparative mitochondrial genomics of snakes: extraordinary substitution rate dynamics and functionality of the duplicate control region
Source: BMC Evol Biol. 2007 Jul 26;7:123. doi: 10.1186/1471-2148-7-123 (PMC1950710; doi:10.1186/1471-2148-7-123)
Supplement: Additional file 4 — All supplementary tables. [file 1471-2148-7-123-S4.pdf]

Additional File 4 (Supplementary Methods)

Supplementary Methods. Laboratory and genome annotation methods.

Total DNA was isolated from frozen (-80C) liver tissue of *Api2* using the Qiagen DNeasy extraction kit and protocol (Qiagen Inc.). Using the Expand Long Template PCR system (Roche Molecular Biochemicals), the mitochondrial genome was amplified in six overlapping fragments with 12 primers (Table S9). In addition, several smaller fragments were also amplified using the BIO-X-ACT Short PCR kit (Bioline) to fill-in otherwise inadequately sequenced regions. Cycling conditions followed the manufacturers' suggestions, with annealing temperatures between 50°C and 55°C, and for 35 cycles.

Positive PCR products were electrophoretically separated and excised from agarose gels, followed by purification using the GeneCleanIII kit (BIO101). Purified PCR products were cloned using either the TopoTA or TopoXL cloning kits (Invitrogen). Plasmids containing amplification fragments were isolated and purified using QIAprep Spin Miniprep kits (Qiagen) and sequenced using M13 primers (flanking the cloning site in the Topo vectors), an array of internal primers (details available upon request), and the CEQ Dye Terminator Cycle Sequencing Quick Start Kit (Beckman-Coulter), and were run on a Beckman CEQ8000 automated sequencer according to the manufacturers' protocols.

Total DNA was extracted from *Api1* using a High Pure PCR Template Preparation Kit (Roche), and amplified into two long overlapping fragments, 8kb and 9kb, using the Expand Long Template PCR Amplification System (Roche) and 4 primers (Table S9). These two fragments overlap in the 16s RNA and COIII genes. Conditions followed the manufacturer's recommendations, with annealing temperatures of 58.4°C (9kb fragment), and 52.2°C (8kb fragment). After electrophoresis as above, PCR products were purified

using the Agarose Gel DNA Purification kit (Mo Bio Laboratory), followed by end phosphorylation, ligation, and shearing in a nebulizer (Invitrogen). Fragments ranging from 1.5-3kb were purified from 0.8% agarose gels using QIAquick Gel Extraction Kit (Qiagen), cloned into pPCR-Script Amp SK(+) vector (Stratagene PCR-Script Amp Cloning Kit), and transformed into XL-10 Gold Kan ultracompetent cells (Stratagene). Bacterial clones containing plasmids with snake mitochondrial inserts were amplified using M13 primers, and the products were purified by QIAquick PCR Purification Kit and sequenced using T3 primer and Big Dye Terminator Sequence Master (PE Biosystems) using standard protocols. The reactions were purified on DyeEx columns (Qiagen), and the DNA sequence was determined using an ABI 3700 automated sequencer.

Total DNA from *Pantherophis* was extracted and amplified using the same protocol and reagents as for *Api1*, but with a different set of four primers (Table S1) yielding 12.5 Kb and 4.5 Kb fragments. These two fragments overlap in the CytB and 16s rRNA genes, and were sequenced following the same protocol as used for *Api1,* with additional internal primers.

Most tRNAs in the raw genome sequences were detected using tRNAscan (Lowe et al. 1997), followed by manual verification. The tRNAs not identified by tRNAscan were identified by their position in the genome and folded manually based on homology. The tRNAs were then used to identify approximate boundaries of protein coding genes, control region, and ribosomal RNAs. Final boundaries of protein coding genes were set based on position of the most plausible first start and last stop codons in each region, including non-canonical signal codons known to operate in vertebrate mitochondrial

genome (Slack et al. 2003). Proteins were also translated to their amino acid sequence, and all amino acid and DNA sequences were compared to the corresponding genes or regions from published snake genomes to verify the annotation.
